# Optimal candidates and surrogate endpoints for HAIC versus Sorafenib in hepatocellular carcinoma: an updated systematic review and meta-analysis

**DOI:** 10.1097/JS9.0000000000001889

**Published:** 2024-08-02

**Authors:** Tengfei Si, Qing Shao, Wayel Jassem, Yun Ma, Nigel Heaton

**Affiliations:** a Department of Hepatobiliary and Pancreatic Surgery, The First Affiliated Hospital, School of Medicine, Zhejiang University, Hangzhou, Zhejiang, People’s Republic of China; b Institute of Liver Studies, King’s College Hospital NHS Foundation Trust, Denmark Hill, London, UK

**Keywords:** HAIC, hepatocellular carcinoma, meta-analysis, sorafenib, surrogate endpoint, systematic review

## Abstract

**Background and aims:**

Hepatic artery infusion chemotherapy (HAIC) has been a long-standing intervention for hepatocellular carcinoma (HCC). Despite positive clinical outcomes, its inclusion in guidelines remains limited due to a lack of evidence-based support. This study aims to identify optimal target populations for HAIC and validate associations between intermediate endpoints with overall survival (OS).

**Methods:**

Following PRISMA guidelines, a comprehensive search was conducted in PubMed, Embase, Cochrane Library, and Web of Science. The primary search strategy was based on medical subject headings terms (MeSH) using ‘Hepatic arterial infusion chemotherapy’, ‘HAIC’, ‘Sorafenib’, ‘Nexavar’, ‘hepatocellular carcinoma’, ‘HCC’, ‘Liver cancer’, combined with free text words. Data extraction, quality assessment, and analysis were performed according to preregistered protocol.

**Results:**

A total of 26 studies, 6456 HCC patients were included for analysis (HAIC, *n*=2648; Sorafenib, *n*=3808). Pooled outcomes revealed that Sorafenib demonstrated better OS only in patients who were refractory to trans-arterial chemoembolization (TACE) (HR=1.32, 95% CI [1.01–1.73]), in other subgroups or overall HCC population HAIC consistently outperformed Sorafenib in patients’ survival. Radiologically, higher response rates in the HAIC group does not necessarily translate into survival improvement, but the hazard ratios (HRs) of 1-year-OS (R^2^=0.41, *P*=0.0044) and 1-year-progression free survival (1y-PFS) (R^2^=0.77, *P*=0.0002) strongly correlated with the patients OS. Meanwhile, larger tumour size (HR=1.86, 95% CI [1.12–3.1, 95%), heavier tumour burden (HR=2.32, 95% CI [1.33–4.02), existence of MVI or EHS (HR=1.65, 95% CI [1.36–2]; HR=1.60, 95% CI [1.19–2.14]), and AFP >400 ng/ml (HR=1.52, 95% CI [1.20–1.92]) were identified as independent risk factors for OS, while HAIC treatment (HR=0.54, 95% CI [0.35–0.82]) and lower BCLC stage (HR=0.44, 95% CI [0.28–0.69]) were potential protective factors for HCC patients.

**Conclusion:**

HAIC monotherapy appears noninferior to Sorafenib in HCC treatment, with potential benefits in specific subgroups. The robust correlation between 1y-OS/1y-PFS and OS, alongside identified risk and protective factors from the present study, offers valuable insights for designing future large prospective studies in this field.

## Introduction

Highlights

- This updated meta-analysis was performed with 6456 HCC patients from 26 studies.
- The potential risk and protective factors of OS for HAIC versus Sorafenib in HCC are unknown from prior meta-analyses and firstly explored in the present study.
- HAIC was consistently associated with higher OS in HCC compared to Sorafenib. However, for patients who were refractory to TACE, Sorafenib demonstrated better OS compared to HAIC.
- Higher response rates (ORR/DCR) after HAIC does not necessarily translate into survival improvement compared to Sorafenib monotherapy, but the intermediate milestone endpoints (1y-OS/1y-PFS) strongly correlated with patients’ OS.
- Higher ECOG score, larger tumour size (>5 cm), heavier tumour burden (>50%), the existence of MVI or EHS and high AFP level (>400 ng/ml) were independent risk factors for patients’ OS. Conversely, HAIC treatment and lower BCLC stage were potentially protective factors.

Hepatocellular carcinoma (HCC) poses a formidable challenge for global heath, often manifesting at an advanced stage with limited therapeutic avenues. As a traditional intervention treatment, hepatic artery infusion chemotherapy (HAIC) has regained new vitality in recent clinical studies. Many centres reported its application in the treatment of advanced HCCs, and most studies demonstrated promising results. Particularly when combined with Sorafenib, HAIC has demonstrated remarkable efficacy^1,2^. However, despite the positive clinical outcomes reported, the absence of evidence-based medical study support has hindered the inclusion of HAIC as a standard HCC treatment option in most guidelines. In the Barcelona Liver Cancer Staging (BCLC), HAIC is not included in the treatment plan for primary liver cancer^3^. The American Association for the Study of Liver Diseases (AASLD) also refrains from recommending HAIC as routine locoregional therapy^4^. The European Association of Liver Diseases (EASL) pointed out the FOLFOX chemotherapy regimen commonly used in HAIC lacks evidence of a survival advantage^5^. In contrast, Sorafenib is widely adopted as a standard of care for advanced HCC in many countries. Since approved by the U.S. Food and Drug Administration (FDA) in 2007, it has received subsequent endorsement in various clinical practice guidelines for HCC management.

So far, the clinical application of HAIC is mainly limited to Asia. The Pan-Asian–adapted European Society for Medical Oncology Guidelines recommended HAIC as one of the first-line options for advanced, nonmetastatic HCC with macrovascular invasion (MVI)^6^. The widespread acceptance of HAIC as one of the treatments for HCC stems primarily its significant locoregional control effect and low systemic toxicity^7^. Our prior investigation showed that HAIC yielded more favourable outcomes in advanced HCC compared to conventional trans-arterial chemoembolization (TACE)^8^. Ongoing research endeavours are dedicated to identifying patients who may benefit from HAIC is required. With additional studies published comparing HAIC with Sorafenib, which suggest that HAIC alone may also achieve similar or even superior therapeutic effects in comparison to Sorafenib monotherapy^9^.

Nevertheless, advanced HCC constitutes a highly heterogeneous group, influenced by factors such as macrovascular invasion and/or metastatic disease, the degree of underlying cirrhosis, and patient’s performance status, all of which can impact patients’ treatment outcome^10^. Consequently, further subclassification is warranted to validate the reliability of conclusion drawn from overall study population. The present study aims to systematically evaluate the clinical outcomes of both therapies in HCC thus to identify: (a) the optimal target populations for HAIC or Sorafenib, and (b) alternative surrogate endpoints which may enhance the design of future clinical trials, along with potential risk/protection factors of OS in patients receiving these treatments.

## Methods

We performed this study according to the Preferred Reporting Items for Systematic Reviews and Meta-Analyses (PRISMA) statement^11^ and assessed the methodological quality by the AMSTAR guidelines^12^. The protocol was registered in PROSPERO international prospective register of systematic reviews.

### Search strategy and selection criteria

Databases including PubMed, Embase, Cochrane Library and Web of Science were searched to collect all available studies about using HAIC and Sorafenib monotherapy in the treatment of advanced HCC. The primary search strategy was based on medical subject headings terms (MeSH), combined with free text words. The following keywords were used as MeSH: ‘Hepatic arterial infusion chemotherapy’, ‘Hepatic artery infusion’, ‘HAIC’, ‘HAI’, ‘Sorafenib’, ‘Nexavar’, ‘SORA’, ‘hepatocellular carcinoma’, ‘HCC’, and ‘Liver cancer’. The original searching cutoff date was 05th August 2023 with an extra updated search was conducted on 15th December 2023. We also checked the reference lists of all identified studies for additional eligible data. Detailed searching strategy was shown in Supplementary Table 3 (Supplemental Digital Content 1, http://links.lww.com/JS9/D228).

Inclusion criteria: (1) Patients: individuals diagnosed with HCC were included; (2) Type of studies: primary research, clinical trials (randomised controlled trials, nonrandomised trials), prospective or retrospective studies (single-centre study, cohort study), and case–controlled studies were considered. Both prospective and retrospective studies were eligible; (3) Outcomes: included studies must report at least one of the following outcomes: overall survival (OS), progression free survival (PFS), radiological response (CR, complete response; PR, partial response; SD, stable disease; PD, progression disease), adverse events and mortality data; (4) The language of the published literature was limited to English only.

Exclusion criteria: (1) Patients without clear diagnosis of HCC or those with metastatic liver cancer; (2) Patients who received combination therapy of HAIC and Sorafenib; (3) In the case of single-centre series with repeated publication or overlapping cases, the research manuscript with more comprehensive data was retained, and (4) letters, editorials, expert opinions, and reviews were excluded to ensure only original data were used.

### Data extraction

Data extraction from each study was performed by two authors (T.F.S. and Q.S.) independently. Patients’ basic characteristics, OS, PFS, details of interventions used, objective response rate (ORR)/disease control rate (DCR), adverse events (Grade 3/4) and mortality data were collected from each study using a predesigned data extraction form (Supplementary Table 1, Supplemental Digital Content 1, http://links.lww.com/JS9/D228 and Table 2, Supplemental Digital Content 1, http://links.lww.com/JS9/D228). In cases of missing information, attempts were made to contact the authors of original articles. Any disagreements during the data extraction process were resolved through discussion or, if necessary, with the involvement of a third reviewer (Y.M.).

### Quality assessment

Two independent reviewers conducted the study quality assessment and risk of bias analysis. The Newcastle–Ottawa Scale (NOS) was utilised to assess the quality of included studies. Regarding the NOS assessment, a maximum of one star could be assigned for each numbered item within the Selection and Exposure categories, while a maximum of two stars would be given for Comparability. Each study was rated from 0 to 9 stars, with 0–3, 4–6, and 7–9 considered low, moderate, and high qualities, respectively, on the NOS scale. The Grading Recommendations Assessment, Development and Evaluation (GRADE) approach was applied to evaluate the certainty of evidence for meta-results^13^. The Guideline Development Tool was accessed from https://www.gradepro.org to create the Summary of Findings table. During the process of quality assessment, disagreement was resolved by discussion or with a third reviewer if necessary (Y.M.).

### Statistical analysis

Review Manager (version 5.3) recommended by Cochrane Collaboration was used to perform the meta-analysis. Hazard ratio (HR) with a 95% CI was selected as effect measure for OS and PFS. Dichotomous variables were tested by risk ratio (RR) with a 95% CI. RRs were calculated using inverse variance method. HRs were calculated using the method suggested by Tierney *et al*.^14^ for incorporating summary time-to-event data into meta-analysis. A random-effect model was used for all calculations. Heterogeneity between studies was tested by *χ*² test (with significance set at *P*>0.1) and *I*² test. Funnel plot was used to investigate publication bias if sufficient studies were available (Supplementary File 14, Supplemental Digital Content 1, http://links.lww.com/JS9/D228). Statistical analysis of patients’ background information was conducted using Fisher’s exact test and *χ* ^2^ test with GraphPad Prism 8.0 (GraphPad Software, Inc.). A *P*-value <0.05 was considered statistically significant. Pooling HRs from multivariate Cox regression were also extracted from each study to identify potential risk or protective factors for OS. Subgroup analysis was performed according to tumour stage, liver function, patient’s treatment history, and other confounding factors which may affect patients’ prognosis.

## Results

### Patients

**Figure 1:**
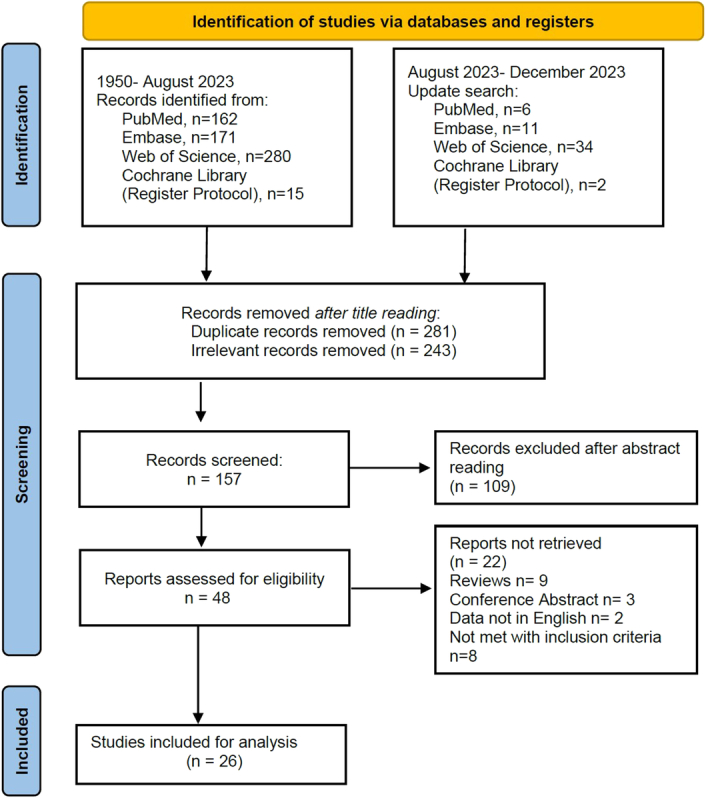
The flowchart of study identification and selection.

The study screening process is provided in Figure 1. A total of 26 studies^9,15–39^ involving 6456 patients were included for the final quantitative analysis. Among whom, 2648 received HAIC treatment, while 3808 patients received Sorafenib monotherapy simultaneously. The participants were mainly from Japan, South Korea, and China. Patients’ tumour characteristics varied across these studies, but the majority exhibited stable liver function (Child-Pugh A-B) (Table 1). To address baseline differences between the HAIC and Sorafenib groups, we summarised eight studies^9,19,25,29–31,36,37^ that employed propensity scoring matching (PSM) design (Supplementary Table 4, Supplemental Digital Content 1, http://links.lww.com/JS9/D228). However, even after PSM, pooled data indicated that in comparison to the Sorafenib group (*n*=562), the HAIC group (*n*=560) continued to have a tendency towards a higher proportion of patients with MVI (46.6 vs. 40.3%, *P*=0.035).

**Table 1:** Baseline characteristics of included studies.

Study	Group	Patients	Sex (M/F)	Age	HBV (Y/N)	Child-Pugh (A/B/C)	BCLC (A/B/C)	AFP (ng/ml)	Tumour size (cm)
Hiramine *et al*. (2011)^27^	HAIC	45	32/13	69.6 (47–84)	11/34	45/0/0	n/a	8.8×10^3^ (0–55.9)	≥5, *n*=22 <5, *n*=23
	Sorafenib	20	17/3	69.6 (44–83)	5/15	n/a	n/a	7.3×10^3^ (0–97.3)	≥5, *n*=8 <5, *n*=12
Jeong *et al*. (2012)^26^	HAIC	21	21/0	51 (33–75)	18/3	10/11/0	0/0/21	≥400, *n*=12 <400, *n*=9	n/a
	Sorafenib	20	11/9	60 (49–75)	13/7	14/6/0	0/0/20	≥400, *n*=13 <400, *n*=7	n/a
Shiozawa *et al*. (2014)^24^	HAIC	77	64/13	67.3±6.8	14/63	49/26/2	0/42/35	≥101, *n*=47 ≤100, *n*=30	n/a
	Sorafenib	47	43/4	69.4±8.2	8/39	39/8/0	0/24/23	≥101, *n*=23 ≤100, *n*=24	n/a
Nemoto *et al*. (2014)^25^	HAIC	8	6/2	74.9±3.4	n/a	4/4/0	n/a	279±418	5.0±2.8
	Sorafenib	12	6/6	80.2±5.4	n/a	10/2/0	n/a	2027±5219	4.2±2.1
Kawaoka *et al*. (2015)^23^	HAIC	136	123/13	67 (30–85)	33/103	136/0/0	0/1/135	415.3 (2.6–1 938 000)	4.5 (1–1.8)
	Sorafenib	41	29/12	69 (30–81)	36/5	41/0/0	0/3/38	208.0 (3–85 632)	4 (1–19)
Fukubayashi *et al*. (2015)^29^	HAIC	128	113/15	67 (65.5±9.3)	33/95	79/49	n/a	18 471.9±77176.9	>5, *n*=66 ≤5, *n*=62
	Sorafenib	72	51/21	69 (68.9±9.8)	33/39	61/11	0/0/72	13 462.3±46460.9	>5, *n*=28 ≤5, *n*=44
Kondo *et al*. (2015)^34^	HAIC	44	32/12	71 (54–84)	1/43	31/13/0	0/16/28	588.5 (3–207 890)	n/a
	Sorafenib	83	74/9	70 (37–88)	15/68	78/5/0	0/58/25	199.7 (1.6–529 490)	n/a
Song *et al*. (2015)^20^	HAIC	50	38/12	54.3±9.9	44/6	45/5/0	0/0/50	<200, *n*=15 ≥200, *n*=35	<10, *n*=22 ≥10, *n*=28
	Sorafenib	60	44/16	55.8±9.0	41/19	47/13/0	0/0/60	<200, *n*=20 ≥200, *n*=38	<10, *n*=31 ≥10, *n*=29
Hatooka *et al*. (2016)^30^	HAIC	65	59/6	67 (46–84)	15/50	65/0/0	0/42/23	415.3 (5–191 500)	4 (1.5–14)
	Sorafenib	58	43/15	70 (50–88)	13/45	58/0/0	0/52/6	449 (5–2446)	3.2 (0.5–19)
Nakano *et al*. (2017)^22^	HAIC	44	33/11	63.4±10.0	n/a	44/0/0	n/a	<1000, *n*=22 ≥1000, *n*=22	7.42±3.33
	Sorafenib	20	17/3	65.4±8.1	n/a	20/0/0	n/a	<1000, *n*=5 ≥1000, *n*=15	7.42±5.42
Yang *et al*. (2017)^21^	HAIC	54	50/4	54.4±11.0	44/10	25/29/0	0/0/54	<400, *n*=23 ≥400, *n*=31	12.5±4.6
	Sorafenib	53	39/14	58.0±9.2	43/10	34/19/0	0/0/53	<400, *n*=23 ≥400, *n*=30	9.2±5.1
Moriguchi *et al*. (2017)^19^	HAIC	32	29/3	65 (40–81)	12/20	32/0/0	n/a	466.1 (5.1–340 140)	7.47 (0–17.9)
	Sorafenib	14	12/2	68 (53–82)	4/10	14/0/0	n/a	416.9 (4.3–211 634)	6.58 (3.27~10.8)
Terashima *et al*. (2017)^32^	HAIC	139	111/28	69 (≥69, *n*=74)	36/103	139/0/0	n/a	≥400, *n*=53 <400, *n*=86	≥5, *n*=51 <5, *n*=88
	Sorafenib	51	45/6	69 (≥69, *n*=27)	16/35	51/0/0	n/a	≥400, *n*=16 <400, *n*=35	≥5, *n*=8 <5, *n*=43
Choi *et al*. (2018)^18^	HAIC	29	25/4	60.3±9.5	21/8	27/2/0	n/a	260.0 (3.6–84 604.6)	<10, *n*=14 ≥10, *n*=15
	Sorafenib	29	27/2	60.2±7.3	18/11	25/4/0	n/a	130.8 (2.0–225 971)	<10, *n*=12 ≥10, *n*=17
Lyu *et al*. (2018)^28^	HAIC	180	160/20	51 (25–77)	156/24	119/61/0	0/4/176	467.3	11.6±3.8
	Sorafenib	232	216/16	51 (16–82)	186/46	159/73/0	0/3/229	478.6	11.7±3.9
Moriya *et al*. (2018)^33^	HAIC	21	16/5	69 (44–88)	n/a	21/0/0	0/21/0	n/a	n/a
	Sorafenib	45	38/7	73 (43–86)	n/a	45/0/0	0/45/0	n/a	n/a
Kodama *et al*. (2018)^15^	HAIC	150	135/15	68	38/112	150/0/0	n/a	464.2	5
	Sorafenib	134	102/32	69	22/112	102/0/0	n/a	448	4.2
Kang *et al*. (2018)^14^	HAIC	95	84/11	55.3±7.6	67/28	59/36/0	0/19/72	12 118.3±34 100.1	8.1±3.7
	Sorafenib	44	37/7	56.6±9.0	34/10	30/14/0	0/17/25	28 144.5±78 708.9	7.4±3.5
Saeki *et al*. (2019)^37^	HAIC	55	42/13	66.7±11.4	n/a	36/19/0	0/55/0	n/a	7.1 (4.0->10.0)
	Sorafenib	78	57/21	72.2±8.5	n/a	60/18/0	0/78/0	n/a	4.0 (2.3–6.2)
Ueshima *et al*. (2020)^31^	HAIC	429	345/84	67.4	91/338	255/174/0	n/a	>400, *n*=218 ≤400, *n*=211	>5, *n*=108 ≤5, *n*=321
	Sorafenib	1346	1081/265	69.9	252/1094	1173/173/0	n/a	>400, *n*=676 ≤400, *n*=670	>5, *n*=275 ≤5, *n*=1071
AHK *et al*. (2021)^17^	HAIC	20	18/2	n/a	n/a	20/0/0	0/0/20	1942.53±5715.44	n/a
	Sorafenib	29	26/3	n/a	n/a	29/0/0	0/0/29	26 102.58±80 097.45	n/a
Ahn *et al*. (2021)^16^	HAIC	38	30/8	53.0±11.6	n/a	27/11/0	n/a	71 341±14 823	n/a
	Sorafenib	35	30/5	58.3±9.5	n/a	24/11/0	n/a	69 745±21 274	n/a
Han *et al*. (2021)^36^	HAIC	151	136/15	56.9±9.7	128/23	n/a	n/a	909.3 (37–11 579)	9.0 (6–12)
	Sorafenib	37	31/6	56.3±10.1	32/5	n/a	n/a	193.2 (12–3909)	8.8 (6.6–12.6)
Zaizen *et al*. (2021)^35^	HAIC	88	61/27	73.8±9.6	6/82	56/32/0	0/71/17	4399±24 315	3.72±3.1
	Sorafenib	243	193/50	72.4±9.5	37/206	196/47/0	0/178/65	7275±49 351	4.2±2.3
Lyu Ning *et al*. (2022)^9^	HAIC	130	115/15	54 (45–61)	120/10	88/42/0	0/5/125	337.8 (28.5–12 902)	11.5±4.5
	Sorafenib	132	123/9	53 (45–62)	114/18	93/39/0	0/9/123	304.2 (15.3–3086.5)	11.0±3.4
Iwamoto *et al.* (2022)^38^	HAIC	418	327/91	68.6±10.9	77/341	418/0/0	0/52/366	27 729.0±154 561.1	8.29±4.60
	Sorafenib	844	677/167	70.1±9.61	154/690	844/0/0	0/305/532	17 735.3±110 114.8	3.59±3.39

### Survival

**Figure 2:**
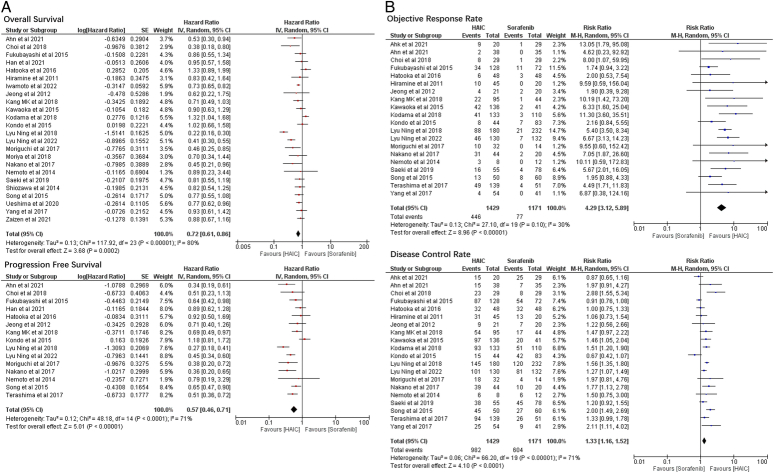
Comparisons of patients’ survival and radiological response between the HAIC group and the Sorafenib group. (A) Forest plots of overall survival (OS) and progression free survival (PFS); (B) Forest plots of objective response rate (ORR) and disease control rate (DCR).

In general, both OS (HR=0.72, 95% CI=0.61–0.86, *I* ^2^=80%) and PFS (HR=0.57, 95% CI=0.46–0.71, *I* ^2^=71%) favoured HAIC compared to Sorafenib (Fig. 2), especially for those with tumours larger than 5 cm, who were chemotherapy naïve or had MVI but without extrahepatic spread (EHS) (Table 2). In patients with Child-Pugh A grade liver function, no significant difference was found in the OS between the two groups, but the HAIC group seemed to have a longer PFS (HR=0.50, 95% CI=0.28–0.91). However, for patients who were refractory to TACE treatment, Sorafenib may offer better OS compared to HAIC (HR=1.32, 95% CI=1.01–1.73). Results from PSM subgroup analysis showed that PFS favouring HAIC over Sorafenib (HR=0.68, 95% CI=0.48–0.98), while no difference existed in OS between the two groups.

**Table 2:** Prognosis and response rate subgroup analysis of HAIC versus sorafenib.

	OS		PFS	
Subgroup	Reference	HR (95% CI)	Reference	HR (95% CI)
Median size ≤5 cm	^15,23,25,27,30,31,35^	0.98 (0.80–1.21)	^25,30^	0.9 (0.51–1.57)
Median size >5 cm	^9,14,18,19,21,22,28,29,36–38,40^	0.6 (0.46–0.78)	^9,14,18,19,22,28,29,36,40^	0.52 (0.40–0.67)
Chemotherapy naive	^9,14,16,18,19,21,26,31,33,37,38^	0.67 (0.56–0.81)	^9,14,16,18,19,26^	0.51 (0.40–0.65)
CTP-A	^15,19,22,23,27,30,33,35,38^	0.82 (0.64–1.05)	^19,22,30^	0.50 (0.28–0.91)
Refractory to TACE	^15,27,30,34^	1.32 (1.01–1.73)	^30,34^	1.1 (0.80–1.52)
EHS (−) MVI (−)	^15,23,31,38^	1.25 (0.86–1.81)	n/a	n/a (n/a)
EHS (+) MVI (−)	^31,38^	1.35 (0.65–2.84)	n/a	n/a (n/a)
EHS (+) MVI (+)	^19,30,31,38^	0.72 (0.44–1.17)	n/a	n/a (n/a)
EHS (−) MVI (+)	^15,18,19,22,23,29,31,38^	0.58 (0.45–0.75)	^18,19,22,29^	0.49 (0.36–0.66)
Propensity score matching	^9,18,24,28–30,35,36^	0.65 (0.38–1.12)	^9,18,28–30,34,36^	0.69 (0.51–0.94)
	1y-OS		1y-PFS	
	Reference	HR (95% CI)	Reference	HR (95% CI)
Median size ≤5 cm	^15,23,25,27,30,31,35^	1.03 (0.81–1.32)	^25,30^	0.90 (0.51–1.57)
Median size >5 cm	^9,14,18,19,21,22,28,29,36–38,40^	0.61 (0.52–0.72)	^9,14,18,19,22,28,29,36,40^	0.54 (0.41–0.70)
Chemotherapy naive	^9,14,16,18,19,21,26,31,33,37,38^	0.66 (0.58–0.76)	^9,14,16,18,19,26^	0.5 (0.39–0.65)
CTP-A	^15,19,22,23,27,30,33,35,38^	0.85 (0.61–1.17)	^19,22,30^	0.50 (0.28–0.91)
Refractory to TACE	^15,27,30,34^	1.36 (1.03–1.79)	^30,34^	0.87 (0.63–1.20)
EHS (−) MVI (−)	^15,23,31,38^	0.90 (0.63–1.30)	n/a	n/a (n/a)
EHS (+) MVI (−)	^31,38^	0.69 (0.58–1.83)	n/a	n/a (n/a)
EHS (+) MVI (+)	^19,30,31,38^	0.74 (0.51–1.06)	n/a	n/a (n/a)
EHS (−) MVI (+)	^15,18,19,22,23,29,31,38^	0.69 (0.51–0.95)	^18,19,22,29^	0.50 (0.34–0.75)
Propensity score matching	^9,18,24,28–30,35,36^	0.78 (0.52–1.16)	^9,18,28–30,34,36^	0.70 (0.51–0.96)
	ORR		DCR	
	Reference	RR (95% CI)	Reference	RR (95% CI)
Median size ≤5 cm	^15,23,25,27,30,31^	5,47 (3.12–9.58)	^15,23,25,27,30,31^	1.28 (1.10–1.50)
Median size >5 cm	^9,14,18,19,21,22,28,29,37,40^	4.32 (2.75–6.78)	^9,14,18,19,21,22,28,29,37,40^	1.50 (1.23–1.84)
Chemotherapy naive	^9,14,16,18,19,21,26,37^	5.89 (3.56–9.76)	^9,14,16,18,19,21,26,37^	1.49 (1.22–1.83)
CTP-A	^15,19,22,23,27,30^	6.23 (3.38–11.46)	^15,19,22,23,27,30^	1.34 (1.09–1.64)
Refractory to TACE	^15,27,30,34^	4.05 (1.42–11.52)	^15,27,30,34^	1.06 (0.77–1.45)
EHS (−) MVI (−)	^15,23^	8.92 (3.70–21.49)	^15,23^	1.49 (1.24–1.80)
EHS (+) MVI (−)	n/a	n/a (n/a)	n/a	n/a (n/a)
EHS (+) MVI (+)	^19,30^	2.85 (0.72–11.36)	^19,30^	1.25 (0.64–2.41)
EHS (−) MVI (+)	^15,18,19,22,23,29^	5.47 (2.28–13.15)	^15,18,19,22,23,29^	1.54 (1.09–2.16)
Propensity score matching	^9,18,28–30^	3.80 (2.00–7.21)	^9,18,28–30^	1.29 (0.98–1.68)

CTP, Child-Turcotte-Pugh; DCR, disease control rate; EHS, extrahepatic spread; HAIC, hepatic arterial infusion chemotherapy; HR, hazard ratio; MVI, major vascular invasion; n/a, not available; ORR, objective response rate; OS, overall survival; PFS, progression free survival; TACE, trans-arterial chemoembolization.

The intermediate milestone survival at 1-year (1y-OS and 1y-PFS) was also explored. The pooled results are generally consistent, HAIC outperformed Sorafenib in terms of 1y-PFS. However, regarding 1y-OS, a statistical difference was found in the subgroup analysis that was not evident in the previous comparison. 1y-OS of patients with EHS but no MVI favoured the HAIC group compared to Sorafenib (HR=0.69, 95% CI=0.51–0.95) (Table 2).

### Radiological response

Regarding radiological response, the HAIC group demonstrated overall advantages compared to Sorafenib in both ORR (RR=4.29, 95% CI=3.12–5.89) and DCR (RR=1.33, 95% CI=1.16–1.52) (Fig. 2). Across subgroup analysis, the objective response relative risk (ORR_RR_) and disease control relative risk (DCR_RR_) of the HAIC group were higher (indicating more patients with ORR/DCR in the HAIC group) than those in the Sorafenib group. Although no difference in DCR was observed between the two groups for patients who were refractory to TACE (RR=1.06, 95% CI=0.77–1.45), the ORR in the HAIC group was still significantly higher compared to the Sorafenib group (RR=4.05, 95% CI [1.42–11.52]). Pooled data from PSM analysis showed similar findings: ORR favoured the HAIC group compared to the Sorafenib group (RR=3.80, 95% CI=2.00–7.21) (Table 2).

### Surrogacy analysis of intermediate endpoints

**Figure 3:**
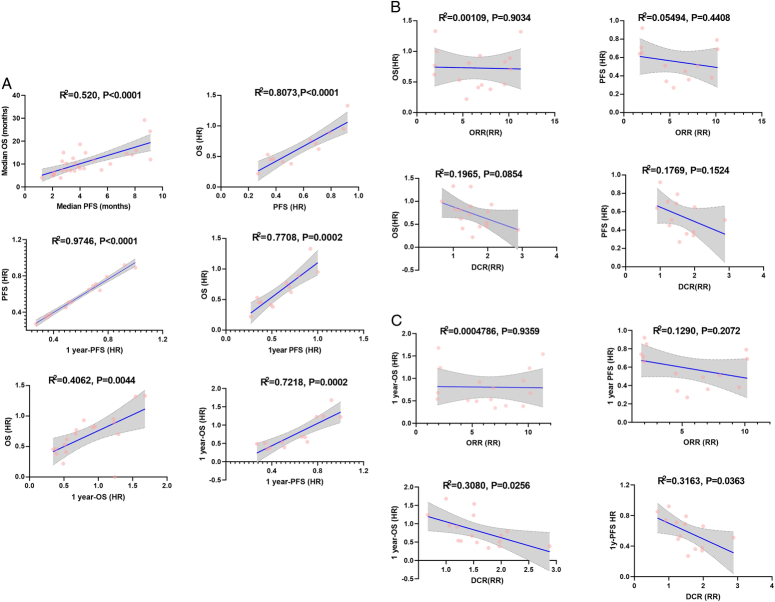
Associations of the treatment effects between intermediate clinical endpoints and HCC patients’ survival after receiving HAIC or Sorafenib (A) Correlation analysis between PFS and OS; (B) Correlation analysis between ORR/DCR and OS/PFS; (C) Correlation analysis between ORR/DCR and 1y-OS/1y-PFS.

Correlation analysis revealed a strong association between PFS_HR_ and OS_HR_, with 1y-PFS_HR_ exhibiting excellent compatibility with PFS_HR_ (R^2^=0.975, 95% CI=0.959–0.996). Both OS_HR_ (R^2^=0.771, 95% CI=0.613–0.965) and 1y-OS_HR_ (R^2^=0.722, 95% CI=0.561–0.954) demonstrated a significant correlation with 1y-PFS_HR_ (Fig. 3A). Although OS_HR_ and PFS_HR_ had a downward trend (indicating longer OS/PFS) with the increasing values of ORR_RR_ and DCR_RR_, no significant correlations were found between ORR_RR_ or DCR_RR_ with OS_HR_/PFS_HR_. Similar results were also observed for PFS analysis (Fig. 3B), while both 1y-OS_HR_ (R^2^=0.3080, *P*=0.026) and 1y-PFS_HR_ (R^2^=0.3163, *P*=0.036) had a statistical association with DCR_RR:_ with increase in DCR_RR_ corresponded to lower HRs of OS and PFS (Fig. 3C).

Seven predefined subgroup analyses, including tumour characteristics, liver function, treatment history, and study design were performed to validate the robustness of correlation analysis results. Overall, the findings remained consistent across subgroups: ORR/DCR showed low association with OS while 1y-OS and 1y-PFS had moderate to high association with OS (Table 3).

**Table 3:** Subgroup surrogacy analysis of intermediate clinical endpoints.

	Correlation of treatment effects (R^2^, 95% CI)				
	ORR_RR_ vs. OS_HR_	DCR_RR_ vs. OS_HR_	1 y-OS_HR_ vs. OS_HR_	1 y-PFS_HR_ vs. OS_HR_	1 y-PFS_HR_ vs. PFS_HR_
Median size ≤5 cm	0.20 (−0.98-0.90)	0.00002 (−0.96-0.97)	0.90 (−0.12-0.999)	n/a	n/a
Median size >5 cm	0.03 (−0.74-0.56)	0.008 (−0.71-0.61)	0.70 (0.44–0.96)	0.87 (0.66–0.99)	0.978 (0.95–0.997)
Chemotherapy naive	0.01 (−0.76-0.65)	0.09 (−0.83-0.51)	0.44 (−0.001-0.92)	0.41 (−0.36-0.96)	0.989 (0.95–0.999)
CTP-A	0.06 (−0.93-0.81)	0.47 (−0.98-0.49)	0.62 (−0.32-0.99)	0.99 (0.991–1.00)	0.96 (0.34–0.99)
Refractory to TACE	0.015 (−0.97-0.95)	0.54 (−0.89-0.99)	0.97 (0.47-0.99)	0.94 (n/a)	n/a
EHS (−) MVI (+)	0.71 (−0.63-0.99)	0.42 (−0.99-0.83)	0.98 (0.75–0.99)	0.94 (n/a)	n/a
Propensity score matching	0.75 (−0.99-0.06)	0.37 (−0.97-0.59)	0.96 (0.85–0.99)	0.85 (0.44–0.99)	0.97 (0.90–0.99)

CTP, Child-Turcotte-Pugh; DCR, disease control rate; EHS, extrahepatic spread; HAIC, hepatic arterial infusion chemotherapy; HR, hazard ratio; MVI, major vascular invasion; n/a, not available; ORR, objective response rate; OS, overall survival; PFS, progression free survival; RR, risk ratio; TACE, trans-arterial chemoembolization.

### Risk/protective factors of OS

**Figure 4:**
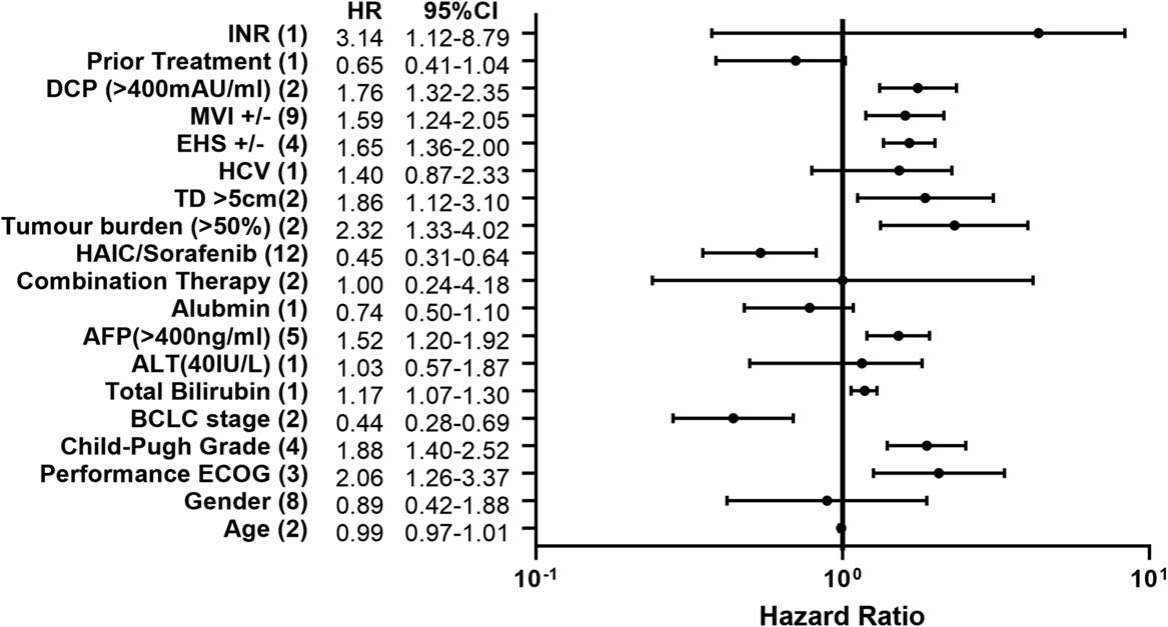
Multivariable HRs of all potential prognostic factors. Prognostic factor (number of studies), Left column: HR and 95% CI from pooled analysis (pooled data if ≥2 studies were included in analysis).

Independent risk and protective factors of OS between the HAIC group and the Sorafenib group were identified in each study. Pooled HRs of multivariate Cox regression indicated that higher ECOG score (HR=2.06, 95% CI=1.26–3.37), larger tumour size (>5 cm) (HR=1.86, 95% CI=1.12–3.10), heavier tumour burden (>50%) (HR=2.32, 95% CI=1.33–4.02), the existence of MVI (HR=1.60, 95% CI [1.19–2.14]) or EHS (HR=1.65, 95% CI [1.36–2.00]) and AFP >400 ng/ml (HR=1.52, 95% CI=1.20–1.92) were independent risk factors for patients’ OS. Conversely, HAIC treatment (HR=0.54, 95% CI=0.35–0.82) and lower BCLC stage (HR=0.44, 95% CI=0.28–0.69) were potentially protective factors (Fig. 4).

### Adverse events

The safety profiles of the two treatments in HCC patients were also compared through meta-analysis. No treatment-related mortality was reported in any of the included studies. Data about grade 3/4 adverse events were documented. Patients in the HAIC group exhibited a higher incidence of elevated ALT (RR=1.29, 95% CI=1.03–1.60), whereas patients receiving Sorafenib monotherapy reported elevated risks for skin complications (RR=0.03, 95% CI=0.01–0.08), diarrhoea (RR=0.37, 95% CI=0.22–0.64), ascites (RR=0.62, 95% CI=0.45–0.85), hepatic encephalopathy (RR=0.31, 95% CI=0.11–0.85), and total serum bilirubin elevation (RR=0.65, 95% CI=0.43–0.97). In terms of other common systematic drug-related side effects such as neutropenia, fatigue, leukopenia, thrombocytopenia, reduced haemoglobin, and hypoalbuminemia, no statistical differences were observed between the two groups (Supplementary Table 5, Supplemental Digital Content 1, http://links.lww.com/JS9/D228).

### Quality assessment and GRADE summary of findings

Most studies were assessed to be of medium-quality (>3 stars, *n*=15) or high-quality (>6 stars, *n*=11). No low-quality studies (≤3 stars) were found to be included. The certainty of evidence after GRADE assessment showed that most findings were of low/very low-quality, findings from comparisons of ORR and OS in patients who are refractory to TACE and comparison of OS in patients with EHS but no MVI presented with moderate quality (Supplementary Table 6, Supplemental Digital Content 1, http://links.lww.com/JS9/D228 and Table 7, Supplemental Digital Content 1, http://links.lww.com/JS9/D228).

## Discussion

Based on 6456 patients from 26 studies, our study showed that HAIC as monotherapy is noninferior to Sorafenib. In specific subgroups, it demonstrates better clinical outcomes, with anticipated longer OS and PFS for individuals with large tumour size (>5 cm), chemotherapy naïve patients and those with MVI but no EHS. Furthermore, HAIC exhibits advantages over Sorafenib in terms of radiological response. Though previous meta-analyses reported similar findings^41,42^, limitations including small sample sizes and high degree of heterogeneity of patient groups which restricted further subgroup analyses based on liver function and treatment history.

Encompassing 26 studies and over 6000 HCC patients, our study stands as the most extensive meta-analysis to date comparing the outcomes of HAIC versus Sorafenib (Supplementary Files 8–13, Supplemental Digital Content 1, http://links.lww.com/JS9/D228 and Supplementary Files15, 16, Supplemental Digital Content 1, http://links.lww.com/JS9/D228). The substantial sample size allows for a comprehensive subgroup-analysis, confirming that patients with Child-Pugh A liver function have better PFS following HAIC compared to those receiving Sorafenib. However, HAIC therapy did not benefit all patients: for those who were refractory to TACE, Sorafenib demonstrated better OS compared to HAIC. This finding contrasts with the outcomes of previous meta-analyses (Liu *et al*.^41^, *n*=417; Zhang *et al*.^43^, *n*=672; Ni *et al*.^44^, *n*=1264; Zhuang *et al*.^45^, *n*=1779); these smaller sized studies generally supported better OS with HAIC compared to Sorafenib in HCC. We believe that in TACE-refractory HCCs, vascular injury due to repeated catheterisation and reduced sensitivity of tumour cells to chemotherapy drugs may provide explanation for the diminished efficacy of HAIC in these patients. Similar findings were also reported in our previous publication^40^, where repeated TACE procedures correlated with a reduced progression-free survival after liver transplant and higher rate of vascular complications.

The advantage of HAIC is the high first pass in the liver allowing higher concentration of chemotherapy drug exposure in the tumour^46^. But there are concerns that chemotherapy from HAIC may not reach effective concentrations in extrahepatic tissues which may also explain the findings from our study: HAIC did not provide greater benefit compared to Sorafenib in the presence of EHS. In such cases, a rational approach might involve adding a systemic chemotherapy agent to HAIC to provide effective systemic control in distant metastatic disease^47,48^. In recent years, numerous studies have reported on the use of combined treatment of HAIC plus Sorafenib for advanced HCC^1,49–51^. While the results of different studies vary, a recent meta-analysis^52^ showed that the antitumour effect of Sorafenib combined with HAIC was better than Sorafenib alone in advanced HCCs with acceptable safety concerns of chemotherapy toxicity.

One novelty of this study was the exploration of surrogate endpoints for effective comparisons of HAIC and Sorafenib. In phase III clinical trials of advanced HCC, OS is usually used as the primary endpoint to verify whether the treatment regimen can truly prolong patient survival. However, in recent years, many studies have included surrogate endpoints such as ORR/PFS in their evaluation. In our study, we found a relatively weak correlation between ORR/DCR and patients’ ultimate survival. A high DCR/ORR does not necessarily translate into clinical benefit. Although radiological response is the most direct indicator of antitumour activity, it is essentially an imaging assessment and may not predict continuing response of patient survival. This potentially explains why, despite overall ORR and DCR in the HAIC group being superior to that from the Sorafenib group, many patients did not exhibit significant improvement in OS after HAIC.

It is noteworthy that during the efficacy comparison between HAIC and Sorafenib, general PFS is significantly correlated with patients’ OS, and the value of 1-year PFS_HR_ was closely related to overall PFS_HR_. A recent meta-analysis^53^ assessing the ability of PFS to predict OS in phase III clinical trials of systemic treatment for advanced HCC, based on 21 randomised controlled trials (RCTs), identified a moderate correlation between PFS and OS. Consequently, the authors proposed a conservative alternative threshold, a PFS_HR_ <0.6, which could be used to predict clinically relevant OS improvement. According to our study, in most comparisons which favour the HAIC group had a PFS_HR_ value below 0.6 and each PFS_HR_ matched OS_HR_ also demonstrated favourable results for HAIC. The robust correlation between OS_HR_ and PFS_HR_ indirectly substantiated the finding that a PFS_HR_ <0.6 could predict clinically relevant OS improvement.

One major limitation in this study was that majority of collected data originated from observational studies and only two RCTs were included. This unavoidably increased the risk of publication bias during the analysis. Future large scale RCTs comparing HAIC with Sorafenib are essential to offer more robust evidence on the effectiveness of HAIC. Such trials should aim to minimise biases and provide comprehensive data that can enhance the reliability of comparative analyses. Additionally, all included studies were from Asia, the results generated may have regional limitations. HCC in Asia is mostly caused by hepatitis B virus (HBV), with many patients having established liver cirrhosis^10^. At the time of diagnosis, they often present with large tumour size or accompanied by portal vein tumour thrombus, resulting in a generally high-risk classification. While in western countries, HCC is mainly caused by chronic hepatitis C virus (HCV) infection and alcohol related liver disease followed by fatty liver disease and diabetes^10^. The diagnostic rate of early-stage HCC is relatively higher with tumours had median or average diameter less than 5 cm when diagnosed, classifying most lesions as low-risk^54^. The IMbrave150 study found that the median OS of atezolizumab combined with bevacizumab in low-risk HCC (BCLC B, no MVI and/or EHS) was more than 2 years, while the median OS of high-risk HCC (VP4) was only 7.6 months^55^. Differences in epidemiology and tumour burden may result in significant differences in the efficacy of the same treatment regimen. Thus, multiple confounding factors need to be carefully considered in clinical and practical applications of HAIC.

This study is different from previous meta-analysis in that it examines independent risk and protective factors for patients’ OS after HAIC or Sorafenib. Factors such as larger tumour size (>5 cm), the presence of MVI or EHS were found to be independent risk factors for patients’ OS, while the choice of HAIC treatment emerged as a protective factor. However, although pooled data indicated the benefit of HAIC for high-risk patients (tumour size >5 cm or with MVI but no EHS), it is crucial to assess the adverse effects of tumour size and MVI on OS when considering HAIC as a treatment option. Tumour size and MVI often reflect the status of tumour blood supply which are closely tied to the efficacy of HAIC. But both factors would also upgrade the BCLC stage of tumours, and pooled HRs suggested that lower BCLC stage may serve as potential protective factor for patients’ OS. Therefore, striking a balance between risk factors and protective factors is pivotal in the consideration of HAIC as the optimal treatment for HCC.

To our knowledge, the present study is the most extensive meta-analysis of HAIC versus Sorafenib in treating HCC. With over 6400 patients across 26 studies, we conducted comprehensive subgroup analysis yielding results generally consistent with those from overall study population. But certain limitations are acknowledged. Firstly, the assessment criteria for radiological response varied among individual studies, potentially introducing bias in the collection and reporting of ORR/DCR. Secondly, considerable heterogeneity exists among different comparisons, partially explainable through the establishment of various subgroups. This diversity may stem from inherent patient differences or variations in HAIC treatment protocols, which currently cannot be definitively attributed due to insufficient data. Thirdly, for single-centre studies covering the same patient population we selected the ones with most comprehensive data, but the inclusion of multicentre studies may lead to some overlap among patients. Finally, most findings from our study generally present with low-quality after GRADE assessment, with a small portion attaining moderate quality. Consequently, high-quality evidence is still lacking.

In conclusion, our findings indicate that some patients may derive benefit from HAIC compared to Sorafenib monotherapy. The strong correlation between 1y-OS/1y-PFS with OS, along with the potential risk and protection factors screened in this study, may inform the design of future large prospective studies focusing on this subject.

## Ethical approval

Not applicable.

## Consent

Not applicable.

## Source of funding

T.F.S. is supported by State Scholarship Fund (201808310051), China Scholarship Council and joint PhD studentship by King’s College London; The Henry Lester Trust; Surgical Funds, King’s College Hospital Charity.

## Author contribution

T.F.S. and N.H.: study design; T.F.S. and Q.S.: manuscript writing; T.F.S., Q.S., and Y.M.: study screening and data analysis; T.F.S., Q.S., Y.M., and N.H.: result discussion; Y.M., W.J., N.H.: proof reading and editing.

## Conflicts of interest disclosure

The authors declare that they have no competing interests.

## Research registration unique identifying number (UIN)

CRD42023458845 (available at https://www.crd.york.ac.uk/prospero/display_record.php?ID=CRD42023458845

## Guarantor

Dr Tengfei Si.

## Data availability statement

All data generated or analysed during this study are included in this manuscript. Further enquiries can be directed to the corresponding author.

## Provenance and peer review

Not commissioned, externally peer-reviewed.

## Supplementary Material
