# Supplementary material for: Optimal candidates and surrogate endpoints for HAIC versus Sorafenib in hepatocellular carcinoma: an updated systematic review and meta-analysis
Source: Int J Surg. 2024 Aug 2;111(1):1203–13. doi: 10.1097/JS9.0000000000001889 (PMC11745638; doi:10.1097/JS9.0000000000001889)
Supplement: Supplementary file 1 [file js9-111-1203-s001.docx]

**Supplementary Files 1-16**

**Supplementary File 1: HAIC & Sorafenib dosage utilized in each study**

| Study | Group | Drugs and dosage | Courses | Interval | Termination |
| --- | --- | --- | --- | --- | --- |
| Hiramine et al (2011) | HAIC | Regimen A (n=25): Daily cisplatin (5 mg/ m^2^) followed by 5-fluorouracil(5-FU,250mg/body); Regimen B (n=12): Daily cisplatin (50 mg/ body), mitomycin C (MMC, 10 mg/body) and epirubicin (EPI, 30 mg/body) on day 1 + cisplatin (5 mg/m^2^) followed by 5-FU (250 mg/body) Regimen C (n=8): Daily cisplatin (50 mg/body), MMC (10 mg/body) and EPI (30 mg/body) | n/a | n/a | Difficulty with continuation of treatment due to disease progression or an adverse effect of grade 3 or higher, a Child-Pugh score ≥10 points or uncontrollable hepatic encephalopathy, intractable ascites, total bilirubin ≥4.0 mg/dl, or a performance status of grade 3 or 4 or worsening of the grade by ≥2 categories |
|  | Sorafenib | Sorafenib was administered orally at 400 mg twice a day. | n/a | n/a |  |
| Jeong et al (2012) | HAIC | Daily cisplatin (7 mg/m^2^) and 5-FU (170 mg/m^2^) on days 1–5 | 2 | 4 weeks | Treatment was stopped in the case of occurrence of grade 4 toxicity |
|  | Sorafenib | Administered as 400 mg b.i.d. doses in 4-week cycles | 1.5 | n/a | Disease progression or intolerable toxicities appeared or the patient refused further treatment |
| Shiozawa et al (2014) | HAIC | Daily cisplatin 10mg/body on days 1-5 and 5-fluorouracil 250mg/body | n/a | 4-8 weeks | Disease progression or unacceptable toxicities occurred |
|  | Sorafenib | Oral doses at 200, 400, or 800 mg/day based on patient physique and liver function | n/a | n/a |  |
| Nemoto et al (2014) | HAIC | 5‐fluorouracil (300 mg/m^2^ on days 1-5 and 8-12) with or without cisplatin (20 mg/m^2^ on days 1 and 8), with interferon-α three times per week for four weeks (5 million units; recombinant interferon‑α, 12 million units; or interferon-α 2b, 3 million units) | n/a | n/a | Disease progression or unacceptable drug toxicity. If the performance status and liver function of patients with PD was preserved, Sora was continued until the occurrence of severe AEs to prevent rapid tumor growth associated with treatment cessation. |
|  | Sorafenib | 200-600 mg/day as an initial dose. In the absence of adverse events, the dose was increased to 400mg twice daily. | n/a | n/a |  |
| Kondo et al (2015) | HAIC | low dose of cisplatin (65 mg/m2) over 30 minutes | 2 (1-11) | 4-6 weeks | Intolerable side effects appeared or disease progression |
|  | Sorafenib | full-dose regimen of sorafenib was 400 mg twice daily | n/a | n/a | Severe adverse events requiring the termination of the treatment appeared, the tumour showed the evidence of remarkable disease progression, or oral administration became intolerable during the follow-up period. |
| Kawaoka et al (2015) | HAIC | 5-FU (300 mg/kg per day) + low dose cisplatin (6 mg/kg per day) or 5-FU (300 mg/kg per day) + 3 × 10^6^ U (3 MU) recombinant IFNα-2b (total dose: 36 M) or 5 MU natural IFN-α (total dose 60 MU) | n/a | 2-4 weeks | Severe side effects, worsening of Eastern Cooperative Oncology Group performance status to 4, aggravation of liver dysfunction or refusal to continue participating in the study. |
|  | Sorafenib | Administered as 400 mg b.i.d. doses and was tapered to 400 mg/day upon the occurrence and severity of adverse events. | n/a | n/a |  |
| Fukubayashi et al (2015) | HAIC | First regimen: daily cisplatin (10mg/body) for 1h followed by 5-FU (250 mg/body); Second regimen: IFN- α (3 million units/body) and 5-FU (500mg/body) | n/a | n/a | Occurrence of radiological and/or symptomatic progression of the HCC was determined, or intolerable adverse events developed |
|  | Sorafenib | Standard daily dose is 800mg; elderly patients (80 years old or older) were started at a reduced daily dose of 400 or 600 mg | n/a | n/a |  |
| Song et al (2015) | HAIC | Cisplatin 60mg/m^2^ for 2h on day 2 and 5-fluorouracil (500mg/m^2^ for 5h on days 1-3) with/without epirubicin (35mg/m^2^ on day1). All patients were given prophylactic anti-emetic treatment with 5-HT_3_ receptor antagonists (ondansetron) | n/a | 3-4 weeks | Both treatments were continued until disease progression, the occurrence of intolerable toxicities, or the patient’s refusal to continue. |
|  | Sorafenib | Administered as 400 mg b.i.d. doses | n/a | 3-4 weeks |  |
| Hatooka et al (2016) | HAIC | 5-FU at 300mg/kg body weight/day + Cisplatin (6mg/kg body weight/day) or 5-FU (300mg/kg body weight/day) + IFN α -2b (total dose 36MU) or natural IFN- α (total dose 60 MU) | n/a | 2-4 weeks | n/a |
|  | Sorafenib | A dose of 400 mg twice daily | n/a | n/a | Until the occurrence of death or meeting one of the following criteria for cessation of therapy: adverse events that required, termination of treatment, deterioration of ECOG PS to 4, worsening liver function, or withdrawal of consent. |
| Terashima et al (2017) | HAIC | Cisplatin injection (20 mg/m^2^/day) +5-fluorouracil (330 mg/m^2^/day) or pegylated interferon-α-2b (1.0 μg/kg) | n/a | 2 weeks | Confirmation of tumour progression, unacceptable toxicity, a patient's refusal of treatment, or death. |
|  | Sorafenib | 400 mg of sorafenib orally twice daily | n/a | n/a |  |
| Nakano et al (2017) | HAIC | A combination of 50mg cisplatin in 5-10ml lipiodol and a continuous infusion of 5FU (1500mg/5days) | n/a | n/a | Discontinued in case of the occurrence of grade 3 or higher adverse effects according to the ECOG classification, except for total bilirubin>3.0mg/dl, platelet count <25*10^9^/l, and leukocyte count <1500/mm^3.^ |
|  | Sorafenib | n/a | n/a | n/a | Discontinued upon development of CTCAE grade 3 or higher adverse events except for a platelet count of <25*10^9^/l and a leukocyte count of <1500/mm^3^ |
| Yang et al (2017) | HAIC | Cisplatin (15mg/m^2^) + 5-FU (50 mg/m^2^) + Epirubicin (30mg/m^2^) | 2 (1-15) | 1 week | Treatment stopped in all grade 3 or grade 4 toxicity cases and was not reinitiated until symptom resolution. |
|  | Sorafenib | 400mg twice a day, orally | n/a | n/a | n/a |
| Moriguchi et al (2017) | HAIC | 4 cycles of intra-arterial infusion of cisplatin (10mg in 1h), +5-FU (250mg in 5h) | 4 | n/a | Disease progression occurred or adverse events impeding continuation developed. |
|  | Sorafenib | Generally commenced at 800mg/day. There were also cases in whom administration was commenced at 400 mg/day, at the primary physician’s discretion | n/a | n/a |  |
| Moriya et al (2018) | HAIC | Cisplatin of 60 mg/m2 over 30 minutes | 3.3±2.0 | 8 weeks | Up to 6 courses or until disease progression or unacceptable adverse events occurred |
|  | Sorafenib | the maximum dose used went up to 800 mg/day. | n/a | n/a | Until disease progression or unacceptable adverse events |
| Choi et al (2018) | HAIC | Cisplatin 60 mg/m^2^ for 2hs on day 2 + 5-FU 500 mg/m^2^ for 5hs on day 1-3. | n/a | 3-4 weeks | Discontinued due to toxicity or death prior to radiological evaluation |
|  | Sorafenib | Generally, 800mg daily oral dose | n/a | n/a |  |
| Lyu et al (2018) | HAIC | Modified FOLFOX6 regimen, including oxaliplatin (130mg/m^2^ infusion for 3hrs on day 1), leucovorin (200mg/m^2^ from 3-5 hrs on day 1) and Fluorouracil (400mg/m^2^ in bolus, and then 2400 mg/m2 continuous infusion 46hrs) | 4 (1-8) | 3 weeks | Continued until intrahepatic lesions progressed or toxicity became unacceptable |
|  | Sorafenib | All patients initially received the standard 400mg dose twice daily. | n/a | n/a | If further dose reduction was required after first dose reduction (400mg once daily) |
| Kodama et al (2018) | HAIC | 5FU at 300mg/kg bodyweight/day and CDDP at 6mg/kg bodyweight/day on days 1-5 and 8-12 plus recombinant IFNα-2b or natural IFNα | n/a | 2-4 weeks | n/a |
|  | Sorafenib | A dose of 400mg twice daily | n/a | n/a | Until death or one of the following criteria was met for cessation of therapy, adverse events that required termination of treatment, deterioration of ECOG PS to 4, worsening liver function, or withdrawal of consent. |
| Kang et al (2018) | HAIC | Cisplatin (25mg/m2 for 12 hrs on days 1-4) and 5-FU (750mg/m2 for 12hrs on days 1-4) with 5-hydroxytryptamine 3 receptor antagonists in all patients. | n/a | 4 weeks | Discontinued when the patient could not tolerate it, severe adverse events occurred, or the cancer progressed. |
|  | Sorafenib | Orally at 400mg twice a day. | n/a | n/a |  |
| Saeki et al (2019) | HAIC | Daily low-dose of FP, including CDDP (10 mg/body), followed by 5-FU (250 mg/body), and isovorin (6.25 mg/body | n/a | 4 weeks | n/a |
|  | Sorafenib | Initial dose of sorafenib was 800 mg, reduced to 400 mg depending on the liver function | n/a | n/a | n/a |
| Ahk et al (2021) | HAIC | Adriamycin (50mg) and cisplatin (50mg) | n/a | n/a | Remission, deterioration of general condition (one patient), or extrahepatic spread (one patient). |
|  | Sorafenib | 400mg twice daily orally. | n/a | n/a |  |
| Ahn et al (2021) | HAIC | Cisplatin (60mg/m^2^) for 1day and 5-FU (500mg/m^2^) for 3 days every 4 weeks. | n/a | 4 weeks | n/a |
|  | Sorafenib | 400mg twice daily, and the dose was adjusted according to adverse events. | n/a | n/a | n/a |
| Han et al (2021) | HAIC | 5-fluorouracil (500 mg/m2 for 5 h on days 1–3) and cisplatin (60 mg/m2 for 2 h on day 2) | n/a | 4 weeks | n/a |
|  | Sorafenib | 400 mg twice daily | n/a | n/a | n/a |
| Zaizen et al (2021) | HAIC | A low dose of 65 mg/m2 cisplatin over 20–40 min. Patients had antiemetic prophylaxis with a 5-HT3 antagonist (granisetron 1 mg) and received adequate hydration and diuretics for protection against cisplatin-induced renal dysfunction. | n/a | 2-3 months | the appearance of tumour progression and/or unacceptable toxicity |
|  | Sorafenib | n/a | n/a | n/a | n/a |
| Lyu et al (2022) | HAIC | the FOLFOX (oxaliplatin 130 mg/m2, leucovorin 200 mg/m2, fluorouracil 400 mg/m2, and fluorouracil 2,400 mg/m2) regimen | 3 (2-4) | 3 weeks | Intrahepatic lesions progressed or toxicity became unacceptable. |
|  | Sorafenib | 400 mg sorafenib orally twice daily | n/a | n/a | If further dose reduction was required after first dose reduction (400mg once daily) |
| Ueshima | HAIC | 64.3% (276 patients), 18.6% (80 patients), and 15.9% (68 patients) received 5FU + CDDP, 5FU + IFN, and CDDP alone, respectively. | n/a | n/a | n/a |
|  | Sorafenib | 400 mg sorafenib orally twice daily |  |  |  |
| Iwamoto et al (2022) | HAIC | Cisplatin (50 mg/body) suspended in 5–10 mL of lipiodol + 5-FU (250 mg bolus injection and 1250 mg continuous injection | n/a | n/a | n/a |
|  | Sorafenib | 2 x 200 mg (tablets) twice a day | n/a | n/a | n/a |

5-fluorouracil, 5-FU; Adverse events, AEs; Cisplatinum, CDDP; Eastern Cooperative Oncology Group, ECOG; Epirubicin, EPI; Hepatic arterial infusion chemotherapy, HAIC; Mitomycin C, MMC; Not available, n/a; Progressive disease, PD;

**Supplementary File 2: Patient selection criteria of included studies for HCC patients**

| Author | Area | Included patients | Excluded patients |
| --- | --- | --- | --- |
| Hiramine et al (2011) | Japan | 1. Advanced HCC unsuitable for surgical resection, liver transplantation, or nonsurgical interventions 2. ECOG performance status 0 or 1 for sorafenib or 0 to 2 for HAIC 3. No other serious medical condition, no history of systematic chemotherapy with sorafenib, no concurrent malignancy of another type, and previously described laboratory findings for sorafenib | 1. Patients who had more than two distant metastases 2. Patients had a distant metastasis of size >1 cm |
| Jeong et al (2012) | Korea | 1. Advanced HCC unsuitable for surgical resection, liver transplantation, or nonsurgical interventions. 2. Age 18-75, have ECOG performance status of 0-2, a Child-Pugh class of A or B, preserved organ function, acceptable blood cell counts, at least one unidimensional measurable lesion. | 1. Patients that had an extrahepatic malignancy 2. Patients that had any other concurrent serious medical conditions 3. Patients had received previous systemic chemotherapy. |
| Shiozawa et al (2014) | Japan | 1. Patients with advanced HCC 2. Sorafenib group: patients in whom medication was possible for one month or longer out of those to whom sorafenib was prescribed once or more. 3. The HAIC group: patients who underwent HAIC one or more times. 4. Both Groups: patients with previous or concomitant treatment with radiofrequency ablation, radiation therapy, TACE, and TAI | 1. Patients with concomitant sorafenib and HAIC |
| Nemato et al (2014) | Japan | 1. Patients with aged≥70 years with histologically or clinically confirmed advanced HCC | 1. Patients who presented with severe vascular invasion. or multiple intrahepatic lesions, 2. Patients with progressive disease (PD) following surgical or locoregional therapy intervention. |
| Kondo et al (2015) | Japan | 1. HCC patients who had been treated with HAIC or sorafenib in routine clinical practice 2. Patients who were not responsive to TACE | 1. Patients who were not treated with TACE 2. TACE-refractory patients with extrahepatic metastasis 3. a Child–Pugh score of 9 or more or Barcelona Clinic Liver Cancer (BCLC) stage A 4. Those who had been treated with HAIC, sorafenib or another type of systemic therapy |
| Kawaoka et al (2015) | Japan | 1. Patients at Class-Pugh class A and with MVI 2. Patients that were refractory to TACE | 1. Patients with extrahepatic metastasis. 2. Patients that had been treated with both HAIC and sorafenib during the follow-up period. |
| Fukubayashi et al (2015) | Japan | 1. Patients with progression of HCC, as documented by PVI, EHS, multiple lesions of both lobes and refractory status to TACE. 2. Eastern Cooperative Oncology Group (ECOG) performance status score of 2 or less. 3. Child-Pugh class A or B, and a life expectancy of at least 12 weeks. | 1. Patients with uncontrollable ascites or hepatic encephalopathy. |
| Song et al (2015) | Korea | 1. Age 18-75 2. Radiologically confirmed PVTT in the main (Vp4), first (Vp3), or second branch (Vp2) of the portal vein. 3. An Eastern Cooperative Oncology Group (ECOG) performance status of 0 or 1 4. Preserved liver function (Child-Pugh score≤7) 5. A white blood cell count≥ 3*109/L or an absolute neutrophil count≥ 1.0*109/L 6. A platelet count≥50 *109/L 7. Patients with extra-hepatic metastases | 1. The presence of another primary tumour and other serious medical conditions e.g., Renal, or cardio-pulmonary insufficiency 2. Patients who were treated with sorafenib in the HAIC group and those who were treated with HAIC in the sorafenib group. |
| Hatooka et al (2016) | Japan | 1. Patients with HCC, Child-Pugh A cirrhosis 2. Free from extrahepatic metastasis and refractory to TACE | 1. Patients who were treated with both HAIC and sorafenib during the follow-up period. |
| Terashima et al (2017) | Japan | 1. HCC patients were judged to be unsuitable for surgery, locoregional therapy, and trans arterial chemoembolization 2. Without prior chemotherapy 3. Without cirrhosis or with Child-Pugh A cirrhosis 4. Presence of intrahepatic lesions 5. Patients with extrahepatic lesions were judged to be eligible for HAIC if the extrahepatic lesions were mild and not prognostic | n/a |
| Nakano et al (2017) | Japan | 1. HCC patients with MVI, without EHS and Child-Pugh class A disease were registered. 2. Eastern Cooperative Oncology Group (ECOG) performance status of 0–2 3. Measurable disease using the Response Evaluation Criteria in Solid Tumours (RECIST) 4. Child-Pugh class A liver function 5. Leukocyte count of ≥2,000/mm3 6. Platelet count of ≥50×109/l 7. Haemoglobin level of ≥8.5 g/dl 8. Serum creatinine level of <1.5 mg/dl 9. No ascites or encephalopathy | n/a |
| Yang et al (2017) | Korea | 1. Age 18-80 years. 2. An Eastern Cooperative Oncology Group performance status of zero to two 3. Child-Pugh Class A or B 4. Advanced HCC according to the BCLC staging classification. 5. Acceptable blood cell counts (absolute neutrophil count≥1.0×109/L and platelet count≥50×109/L) 6. Vascular access to the lesion for implantation of a chemo port | 1. Evidence of hepatic decompensation 2. Concurrent serious medical condition(s), eg. underlying cardiac or renal disease 3. Other concurrent primary malignancy 4. Another chemotherapy such as hepatic arterial infusion chemotherapy (HAIC) or systemic chemotherapy 5. Adjuvant MET chemotherapy or sorafenib after curative treatment. 6. Absence of an intrahepatic lesion |
| Moriguchi et al (2017) | Japan | 1. Clinically diagnosed HCC with Vp3 or Vp4 as determined by blood testing (viral markers, tumour markers) and dynamic contrast-enhanced computed tomography or magnetic resonance imaging 2. Child-Pugh A 3. Eastern Cooperative Oncology Group (ECOG) performance status (PS) of 0 or 1 4. White blood cell count ≥2,000/μL, haemoglobin ≥8.0 g/dL, and platelet count ≥6.0 × 104/μL 5. Inability to undergo surgical resection, or refractory to or unsuitable for transarterial chemoembolization 6. No concomitant use of other treatments | n/a |
| Moriya et al (2018) | Japan | 1. Advanced HCC with Child-Pugh class A hepatic functional reserve 2. Chemo-naïve 3. Refractory to TACE or had distinct extrahepatic metastasis lesions | n/a |
| Choi et al (2018) | Korea | 1. Age 18–70 years 2. First diagnosed advanced HCC or TACE- refractory advanced HCC with portal vein tumour thrombosis (Vp3 or Vp4 PVTT) 3. An Eastern Cooperative Oncology Group (ECOG) performance status of 0 or 1 4. Child–Turcotte–Pugh (CTP) score of 5–7 5. Appropriate bone marrow function such as white blood cell count ≥ 4.0 × 103/ μL, platelet count ≥ 60 × 103/μL, absolute neutrophil count (ANC) ≥ 1.5 × 103/μL 6. Serum creatinine level of ≤ 1.5 mg/dL 7. No blood coagulation disorders 8. No extrahepatic primary malignancy or metastasis | 1. Other concurrent serious medical condition(s) such as underlying cardiac or renal disease, infectious disease 2. Pregnant women, lactating women 3. Other chemotherapy treatments such as systemic chemotherapy 4. History of gastrointestinal bleeding within 2 weeks of enrolment |
| Lyu et al (2018) | China | 1. An adequate blood/bone marrow (leukopenia count >3.0 × 109/L, haemoglobin >8.0 g/L, and platelet count >60 × 109/L) 2. Liver (alanine aminotransferase [ALT] and aspartate aminotransferase [AST] <5 times the upper limit of the normal range, albumin >2.8 g/L, total bilirubin <2.8 g/L) 3. Renal (serum creatinine <1.5 times the upper limit of the normal range) and coagulation (prothrombin time <6 s) function 4. A controlled arterial hypertension. | 1. Child-Pugh score of C. 2. Eastern Cooperative Oncology Group-performance status (ECOG-PS) >2 3. At least one incomplete cycle of HAIF or four weeks of sorafenib 4. Without intrahepatic measurable lesions 5. Lack of the first image screen 6. A secondary malignancy 7. Treated with oxaliplatin or fluorouracil chemotherapy before and lost to follow-up |
| Kodama et al (2018) | Japan | 1. No extrahepatic metastasis 2. Child-Pugh A status 3. Not having received treatment with either HAIC or sorafenib during the course | n/a |
| Kang et al (2018) | Korea | 1. Age 20 to 80 years 2. PVT, lymph node, and distant metastasis 3. An Eastern Cooperative Oncology Group performance status 1 or 2 4. Preserved liver function below Child–Pugh grade B 5. Intermediate-stage HCC, which is not eligible for transcatheter arterial chemoembolization. | 1. Other malignant tumours except HCC 2. Serious medical condition such as cardiopulmonary or renal insufficiency 3. previous systemic intravenous chemotherapy 4. <2 cycles of HAIC |
| Saeki et al (2019) | Japan | 1. HCC with macrovascular invasion (MVI) and extra-hepatic spread (EHS) 2. Untreatable by loco-regional therapies 3. Either HAIC or sorafenib was administered as a first line treatment | 1. Patient records without computed tomography (CT) within 1 month of starting HAIC or sorafenib were excluded |
| Ueshima et al (2020) | Japan | 1. Aged 20 years or older with advanced HCC who received HAIC or sorafenib treatment | 1. Patients who received combination therapies with HAIC or sorafenib (e.g., sorafenib combined with TACE, HAIC combined with sorafenib, sequential therapy of HAC and sorafenib, or HAIC combined with radiation therapy |
| AHK et al (2021) | Egypt | 1. Patients with compensated liver disease (Child-Pugh score A) due to chronic hepatitis C-related cirrhosis 2. Patients with HCC (single or multiple focal lesions) at an advanced stage due to partial or segmental portal vein thrombosis (Barcelona Clinic Liver Cancer [BCLC] stage C). | n/a |
| Ahn et al (2021) | Korea | 1. Patients with advanced HCC were treated with sorafenib or HAIC 2. Patients with main PVTT (tumour thrombosis in the main portal trunk on radiographic images such as computed tomography or magnetic resonance images with dynamic enhance) | n/a |
| Han et al (2021) | Korea | n/a | 1. Patients with diffuse or multifocal bi-lobal tumours 2. Patients with extrahepatic metastasis |
| Zaizen et al (2021) | Japan | For HAIC:   1. Diagnosed with intrahepatic advanced HCC   For Sorafenib:   1. Eastern Cooperative Oncology Group performance status of 0–1 2. measurable disease using the Response Evaluation Criteria in Solid Tumours 3. Child-Pugh class A or B 4. Leukocyte count ≥ 2000/mm^3^ 5. Platelet count ≥ 50 × 10^9^/L 6. Haemoglobin level ≥ 8.5 g/dL 7. Serum creatinine level < 1.5 mg/dL 8. No ascites or encephalopathy | 1. with extrahepatic metastasis or with BCLC stage A lesion |
| Lyu Ning et al (2022) | China | 1. Age ≥ 18 years with a locally advanced or unresectable HCC confirmed histologically or with cirrhosis diagnosed clinically. 2. A dominant mass in the liver with or without extrahepatic metastasis, which was defined as up to three metastatic lesions in up to two organs with the largest diameter of ≤ 3 cm. 3. Disease that is unsuitable for surgery, ablation, or trans arterial chemoembolization, or progressive disease after such therapies, no prior systemic options performed 4. Eastern Cooperative Oncology Group Performance Status (ECOG-PS) of 0-2, Child-Pugh grade ≤ 7 5. Adequate hematologic function: white blood cell count ≥ 3.0 × 109/L, neutrophils ≥ 1.5 × 109/L, platelets ≥ 50 × 109/L, haemoglobin ≥ 100 g/L 6. Adequate hepatic function: serum AST < 5 times the upper limit of normal, serum ALT < 5 times the upper limit of normal, total serum bilirubin < 51 μmol/L, serum albumin ≥ 2.8 g/dL 7. Adequate coagulation function: prothrombin time-international normalized ratio ≤ 2.3 or prothrombin time < 6 seconds above control 8. Adequate renal function: serum creatinine < 110 μmol/L | n/a |
| Iwamoto et al (2022) | Japan | 1. Patients diagnosed with HCC; 2. Patients with preserved liver function, typically indicated by Child-Pugh class A. 3. Patients who received HAIC with New-FP (FUDR). 4. Availability of baseline clinical and laboratory data. 5. Adequate follow-up data until death or the final follow-up visit. 6. Patients who provided informed consent to participate in the study. | n/a |

**Supplementary File 3: Searching Strategies**

| ***Name of database*** | 1. ***Time*** span | ***Search strategy*** | ***Items*** |
| --- | --- | --- | --- |
| The Cochrane Central Register of Controlled Trials (CENTRAL) in The Cochrane Library | Issue 6，2023 | #1 ((hepatocellular or liver cell or hepatic) and (carcinoma* or tumo* or cancer)) or HCC or hepatoma*:ti,ab,kw (Word variations have been searched)  #2 ((liver or hepatic or Intra) and (infusion)) or HAIC or HAI: ti,ab,kw (Word variations have been searched)  #3 (sorafenib or Nexavar):ti,ab,kw (Word variations have been searched)  #4 MeSH descriptor: [Carcinoma, Hepatocellular] explode all trees  #5 MeSH descriptor: [arterial infusion] explode all trees  #6 #1 or #4  #7 #2 or #5  #8 #3  #9 #6 and #7 and #8 | n=15 |
|  | August 2023-December 2023 |  | n=2 |
| PubMed | 1950- August 2023 | ((((((((((carcinoma, hepatocellular[MeSH Terms]) OR hepatocellular carcinoma[Title/Abstract]) OR HCC[Title/Abstract]) OR hepatocarcinoma[Title/Abstract]) OR hepatomas[Title/Abstract]) OR liver carcinoma[Title/Abstract]) OR liver cancer[Title/Abstract]) OR liver cell carcinoma[Title/Abstract])) AND ((((hepatic arterial infusion chemotherapy[Title/Abstract]) OR hepatic arterial infusion[Title/Abstract]) OR HAIC[Title/Abstract]) OR HAI[Title/Abstract])) AND (Sorafenib[Title/Abstract] OR Sorafenib Monotherapy[Title/Abstract])) | n=162 |
|  | August 2023-December 2023 |  | n=6 |
| EMBASE | 1966- December 2023 | (((hepatocellular AND ('carcinoma'/exp OR carcinoma) OR hcc OR 'hepatocarcinoma'/exp OR hepatocarcinoma OR hepatomas OR 'liver'/exp OR liver) AND ('carcinoma'/exp OR carcinoma) OR 'liver'/exp OR liver) AND ('cancer'/exp OR cancer) OR 'liver'/exp OR liver) AND ('cell'/exp OR cell) AND ('carcinoma'/exp OR carcinoma) AND ((hepatic AND arterial AND ('infusion'/exp OR infusion) AND ('chemotherapy'/exp OR chemotherapy) OR hepatic) AND arterial AND ('infusion'/exp OR infusion) OR haic OR hai) AND Sorafenib AND [abstracts]/lim AND [embase]/lim AND [english]/lim | n=171 |
|  | August 2023-December 2023 |  | n=11 |
| Web of Science | 1945- December 2023 | (TS= ("carcinoma, hepatocellular" OR "hepatocellular carcinoma" OR HCC OR "hepatocarcinoma" OR hepatomas OR "liver carcinoma" OR "liver cancer" OR "liver cell carcinoma")) AND (TS=("hepatic arterial infusion chemotherapy" OR "hepatic arterial infusion" OR HAIC OR HAI)) AND (TS=("Sorafenib" OR "Sorafenib Monotherapy" OR" Nexavar ")) | n=280 |
|  | August 2023-December 2023 |  | n=34 |

**Supplementary File 4: Baseline Characteristics of HCC patients with propensity score matching**

|  | Overall | |  |
| --- | --- | --- | --- |
|  | HAIC | Sorafenib | P value |
| Sex ^a^ |  |  |  |
| M | 429 | 444 | 0.2308 |
| F | 78 | 65 |  |
| Aetiology ^a^ |  |  |  |
| HBV | 320 | 315 | 0.8726 |
| HCV | 137 | 145 |  |
| Others | 50 | 49 |  |
| Child-Pugh ^b^ |  |  |  |
| A | 383 | 397 | 0.4119 |
| B | 146 | 135 |  |
| Treatment History ^c^ |  |  |  |
| Y | 149 | 151 | 0.9275 |
| N | 245 | 245 |  |
| BCLC stage ^d^ |  |  |  |
| B | 140 | 141 | 0.9431 |
| C | 325 | 324 |  |
| Major Vessel Invasion ^e^ |  |  |  |
| Y | 261 | 226 | **0.035** |
| N | 299 | 336 |  |
| Extrahepatic metastasis^f^ |  |  |  |
| Y | 137 | 149 | 0.4514 |
| N | 423 | 413 |  |

References: a: 9, 18, 24, 28, 29, 30, 35, 36; b: 9, 18, 24, 28, 29, 30, 35; c: 9, 18, 24, 28, 30; d: 9, 24, 28, 30, 35

e: 9, 18, 24, 28, 29, 30, 35, 36; f: 9, 18, 24, 28, 29, 30, 35, 36

**Supplementary File 5: Grade III-IV adverse events comparisons between HAIC and Sorafenib**

| **Adverse Event** | **Studies** | **Participants** | **Risk Ratio, 95%CI** |
| --- | --- | --- | --- |
| Skin disorder | 9 | 2963  HAIC=1046; Sora=1917 | 0.03 [0.01, 0.08] |
| Elevated ALT | 8 | 1247  HAIC=634; Sora=613 | 1.29 [1.03-1.06] |
| Diarrhoea | 7 | 1017  HAIC=474; Sora=543 | 0.37 [0.22-0.64] |
| Ascites | 6 | 1013  HAIC=536; Sora=477 | 0.62 [0.45-0.64] |
| Neutropenia | 6 | 854  HAIC=409; Sora=445 | 1.70 [0.71-4.11] |
| Hepatic encephalopathy | 5 | 311  HAIC=177; Sora=134 | 0.31 [0.11-0.85] |
| Elevated total bilirubin | 5 | 691  HAIC=384; Sora=307 | 0.65 [0.43-0.97] |
| Fatigue | 5 | 915  HAIC=413; Sora=502 | 0.85 [0.45-1.61] |
| Thrombocytopenia | 5 | 915  HAIC=413; Sora=502 | 1.56 [0.58-4.16] |
| Leukopenia | 4 | 989  HAIC=505; Sora=484 | 0.96 [0.54-1.70] |
| Reduced hemoglobin | 3 | 862  HAIC=461; Sora=401 | 0.56 [0.16-1.99] |
| Hypoalbuminemia | 3 | 694  HAIC=318; Sora=376 | 0.83 [0.68-1.01] |

**Supplementary File 6: Critical appraisal using the Newcastle-Ottawa Quality Assessment Scale for Cohort Study**

|  | Selection | | | | Comparability | Outcome | | | Total |  |  |  |  |  |  |  |  |
| --- | --- | --- | --- | --- | --- | --- | --- | --- | --- | --- | --- | --- | --- | --- | --- | --- | --- |
| Resource | Representativeness  of the exposed cohorts | Selection of the  non-exposed cohorts | Ascertainment of exposure | Demonstration that outcome of interest |  | Ascertainment of outcome | Length of follow-up | Adequacy of follow-up |  |  |  |  |  |  |  |  |  |
| Lyu 2022 | ★ | ★ | ★ | - | ★★ | ★ | ★ | ★ | ★★★★★★★★ | |  |  |  |  |  |  |  |
| Kang 2019 | ★ | ★ | ★ | - | ★ | ★ | ★ | ★ | ★★★★★★★ | |  |  |  |  |  |  |  |
| Kodama 2018 | ★ | ★ | ★ | ★ | - | ★ | ★ | - | ★★★★★★ | |  |  |  |  |  |  |  |
| Ahn 2021 | ★ | ★ | ★ | - | ★ | ★ | - | - | ★★★★★ | |  |  |  |  |  |  |  |
| Ahk 2021 | ★ | - | ★ | - | ★ | ★ | ★ | - | ★★★★★ | | - | ★ | - | ★★ | ★ | ★ | ★ |
| Choi 2018 | ★ | ★ | ★ | ★ | ★★ | ★ | - | ★ | ★★★★★★★★ | |  |  |  |  |  |  |  |
| Moriguchi 2017 | ★ | ★ | ★ | - | ★ | ★ | - | - | ★★★★★ | |  |  |  |  |  |  |  |
| Song 2015 | ★ | - | ★ | - | ★ | ★ | ★ | - | ★★★★★ | |  |  |  |  |  |  |  |
| Yang 2017 | ★ | ★ | ★ | ★ | ★★ | ★ | ★ | - | ★★★★★★★★ | |  |  |  |  |  |  |  |
| Nakano 2017 | ★ | ★ | ★ | - | ★ | ★ | - | ★ | ★★★★★★ | |  |  |  |  |  |  |  |
| Kawaoka 2015 | ★ | - | ★ | ★ | ★★ | ★ | ★ | ★ | ★★★★★★★★ | |  |  |  |  |  |  |  |
| Shiozawa 2014 | ★ | ★ | ★ | ★ | ★★ | ★ | - | - | ★★★★★★★ | |  |  |  |  |  |  |  |
| Nemoto 2014 | ★ | ★ | ★ | - | - | ★ | ★ | - | ★★★★★ | |  |  |  |  |  |  |  |
| Jeong 2012 | ★ | - | ★ | ★ | - | ★ | ★ | ★ | ★★★★★★ | |  |  |  |  |  |  |  |
| Hiramine 2011 | ★ | ★ | ★ | ★ | ★ | ★ | ★ | ★ | ★★★★★★★★ | |  |  |  |  |  |  |  |
| Lyu 2018 | ★ | ★ | ★ | - | ★★ | ★ | - | ★ | ★★★★★★★ | |  |  |  |  |  |  |  |
| Fukubayashi 2017 | ★ | ★ | ★ | - | ★★ | ★ | ★- | ★ | ★★★★★★★★ | |  |  |  |  |  |  |  |
| Hatooka 2016 | ★ | ★ | ★ | - | ★★ | ★ | - | - | ★★★★★★ | |  |  |  |  |  |  |  |
| Ueshima 2020 | ★ | ★ | ★ | ★ | ★★ | ★ | ★ | - | ★★★★★★★★ | |  |  |  |  |  |  |  |
| Terashima 2017 | ★ | - | ★ | - | ★★ | ★ | ★ | ★ | ★★★★★★★ | |  |  |  |  |  |  |  |
| Moriya 2018 | ★ | ★ | ★ | ★ | ★ | ★ | ★ | - | ★★★★★★★ | |  |  |  |  |  |  |  |
| Kondo 2015 | ★ | ★ | ★ | ★ | ★★ | ★ | ★ | - | ★★★★★★★★ | |  |  |  |  |  |  |  |
| Zaizen 2021 | ★ | ★ | ★ | ★ | ★★ | ★ | ★ | - | ★★★★★★★★ | |  |  |  |  |  |  |  |
| Han 2021 | ★ | - | ★ | - | ★★ | ★ | ★ | - | ★★★★★★ | |  |  |  |  |  |  |  |
| Saeki 2019 | ★ | ★ | ★ | ★ | - | ★ | ★ | - | ★★★★★★ | |  |  |  |  |  |  |  |
| Iwamoto 2022 | ★ | ★ | ★ | ★ | ★★ | ★ | ★ | - | ★★★★★★★★ | |  |  |  |  |  |  |  |
|  |  |  |  |  |  |  |  |  |  | |  |  |  |  |  |  |  |

**Supplementary File 7: GRADE Summary of Findings**

| **Outcomes** | **№ of participants (studies) Follow-up** | **Certainty of the evidence (GRADE)** | **Relative effect (95% CI)** | **Anticipated absolute effects** | |
| --- | --- | --- | --- | --- | --- |
|  |  |  |  | **Risk with [Sorafenib]** | **Risk difference with [HAIC]** |
| Overall Survival assessed with: HR | (24 observational studies) | ⨁◯◯◯ Very low^a,b,c,d,e^ | HR 0.72 (0.61 to 0.86) | 0 per 1,000 | **-- per 1,000** (-- to --) |
| General PFS assessed with: HR | (15 observational studies) | ⨁⨁◯◯ Low^a,b,c^ | HR 0.57 (0.46 to 0.71) | 0 per 1,000 | **-- per 1,000** (-- to --) |
| ORR assessed with: RR | 2600 (20 observational studies) | ⨁⨁⨁◯ Moderate^a,e,f^ | RR 4.29 (3.12 to 5.89) | 4 per 1,000 | **14 more per 1,000** (9 more to 21 more) |
| DCR assessed with: RR | 2600 (20 observational studies) | ⨁⨁◯◯ Low^a,e,f^ | RR 1.33 (1.16 to 1.52) | 61 per 1,000 | **20 more per 1,000** (10 more to 32 more) |
| OS in patients with chemotherapy naive assessed with: HR | (14 observational studies) | ⨁⨁◯◯ Low^a,c,d^ | HR 0.67 (0.56 to 0.81) | 0 per 1,000 | **-- per 1,000** (-- to --) |
| OS in patients with Child-Pugh A liver function assessed with: HR | (9 observational studies) | ⨁◯◯◯ Very low^a,c,d,e^ | HR 0.82 (0.64 to 1.05) | 0 per 1,000 | **-- per 1,000** (-- to --) |
| OS of patients refractory to TACE Treatment assessed with: HR | (4 observational studies) | ⨁⨁⨁◯ Moderate^a,c^ | HR 1.32 (1.01 to 1.73) | 0 per 1,000 | **-- per 1,000** (-- to --) |
| OS with EHS (-) MVI (-) assessed with: HR | (5 observational studies) | ⨁⨁◯◯ Low^a,c,d^ | HR 1.25 (0.86 to 1.81) | 0 per 1,000 | **-- per 1,000** (-- to --) |
| OS with EHS (+) MVI (-) assessed with: HR | (2 observational studies) | ⨁⨁◯◯ Low^a,c,d^ | HR 1.35 (0.65 to 2.84) | 0 per 1,000 | **-- per 1,000** (-- to --) |
| OS with EHS (+) MVI (+) assessed with: HR | (4 observational studies) | ⨁⨁◯◯ Low^a,c,d^ | HR 0.72 (0.44 to 1.17) | 0 per 1,000 | **-- per 1,000** (-- to --) |
| OS with EHS (-) MVI (+) assessed with: HR | (9 observational studies) | ⨁⨁⨁◯ Moderate^a,c^ | HR 0.58 (0.45 to 0.75) | 0 per 1,000 | **-- per 1,000** (-- to --) |
| 1y OS assessed with: HR | (24 observational studies) | ⨁⨁◯◯ Low^a,b,c^ | HR 0.75 (0.64 to 0.89) | 0 per 1,000 | **-- per 1,000** (-- to --) |
| 1y-PFS assessed with: HR | (15 observational studies) | ⨁⨁◯◯ Low^a,b,c^ | HR 0.57 (0.47 to 0.70) | 0 per 1,000 | **-- per 1,000** (-- to --) |
| PSM OS assessed with: HR | (8 observational studies) | ⨁◯◯◯ Very low^a,b,c,d^ | HR 0.65 (0.38 to 1.12) | 0 per 1,000 | **-- per 1,000** (-- to --) |
| PSM PFS assessed with: HR | 0 cases 0 controls (7 observational studies) | ⨁⨁◯◯ Low^a,b,c^ | HR 0.69 (0.51 to 0.94) | **Low** | |
|  |  |  |  | 0 per 1,000 | **-- per 1,000** (-- to --) |
| PSM 1y-OS assessed with: HR | (8 observational studies) | ⨁⨁◯◯ Low^a,b,c^ | HR 0.63 (0.36 to 1.12) | 0 per 1,000 | **-- per 1,000** (-- to --) |
| PSM 1y-PFS assessed with: HR | (7 observational studies) | ⨁⨁◯◯ Low^a,b,e^ | HR 0.58 (0.45 to 0.74) | 0 per 1,000 | **-- per 1,000** (-- to --) |
| ***The risk in the intervention group** (and its 95% confidence interval) is based on the assumed risk in the comparison group and the **relative effect** of the intervention (and its 95% CI).  **CI:** confidence interval; **HR:** hazard ratio; **RR:** risk ratio | | | | | |
| **GRADE Working Group grades of evidence** **High certainty:** we are very confident that the true effect lies close to that of the estimate of the effect. **Moderate certainty:** we are moderately confident in the effect estimate: the true effect is likely to be close to the estimate of the effect, but there is a possibility that it is substantially different. **Low certainty:** our confidence in the effect estimate is limited: the true effect may be substantially different from the estimate of the effect. **Very low certainty:** we have very little confidence in the effect estimate: the true effect is likely to be substantially different from the estimate of effect. | | | | | |

a. Most studies were observational, not RCT. There existed unavoidable selection bias

b. High heterogeneity was observed in the comparison.

c. Some HRs were not directly collected from each study but calculated using time-to-event data

d. OS of HCC could also be affected by sequential therapies.

e. Obvious publication bias was noticed from funnel plot.

f. Some studies using different criteria to assess patients’ treatment response

**TREATMENT EFFECTS**

**Supplementary File 8A-D Radiological Response**

**Supplementary Figure 8A Forest plot about the risk ratio of complete response (CR) in the comparison between the HAIC group and the Sorafenib group.**

**
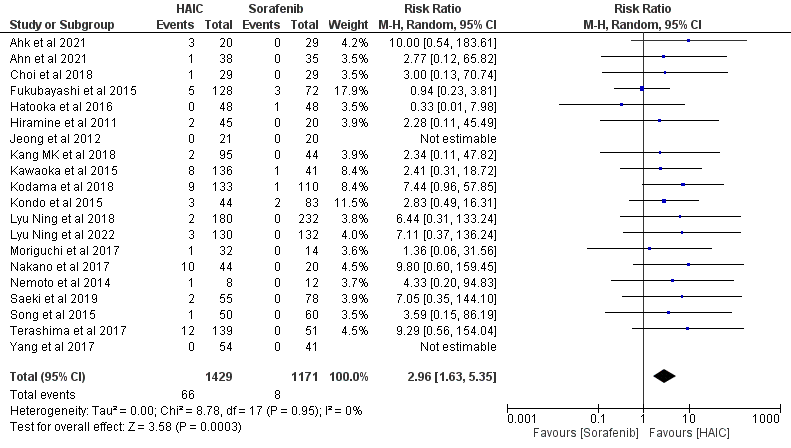
**

**Supplementary Figure 8B Forest plot about the risk ratio of partial response (PR) in the comparison between the HAIC group and the Sorafenib group.**

**
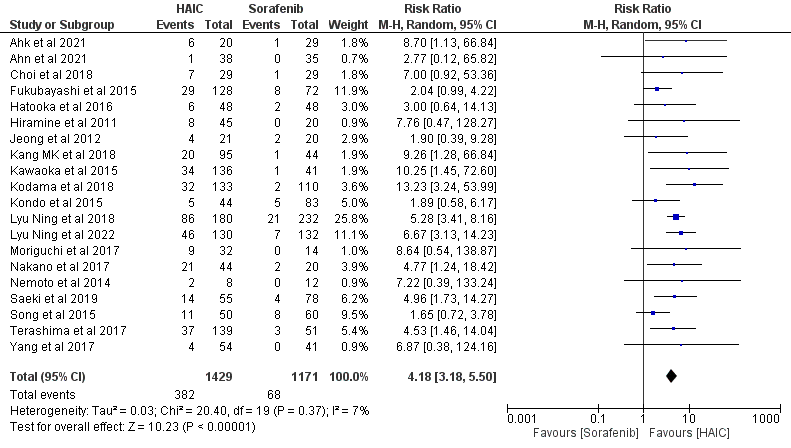
**

**
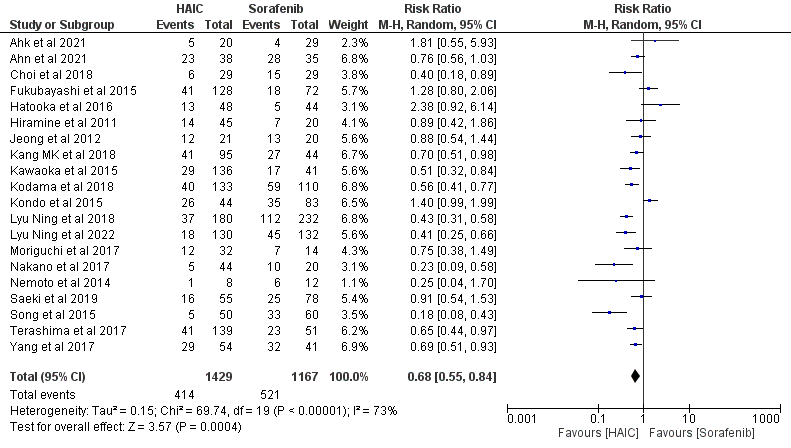
Supplementary Figure 8C Forest plot about the risk ratio of progressive disease (PD) in the comparison between the HAIC group and the Sorafenib group.**

**Supplementary Figure 8D Forest plot about the risk ratio of stable disease (SD) in the comparison between the HAIC group and the Sorafenib group.**

**
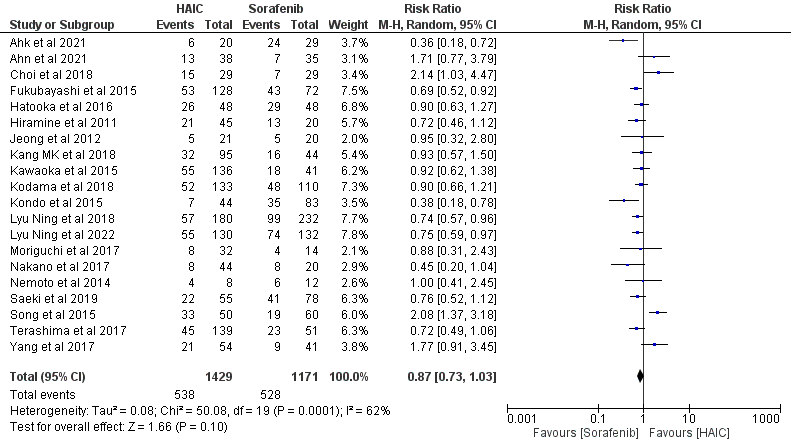
**

**Supplementary notation 8E: Assessment Criteria of Radiological Response**

Both Response Evaluation Criteria in Solid Tumours (RECIST) or the modified RECIST (mRECIST) were used for response assessment.

**Supplementary File 9 A-C Subgroup analysis of overall survival based on patients’ liver function and treatment history**

**
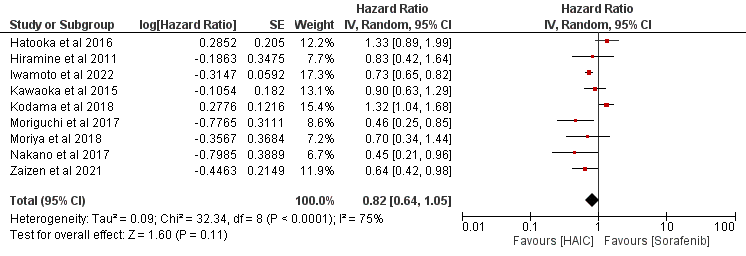
Supplementary Figure 9A Forest plot about the hazard ratio of overall survival (OS) in HCC patients with Child-Pugh A liver function**

**
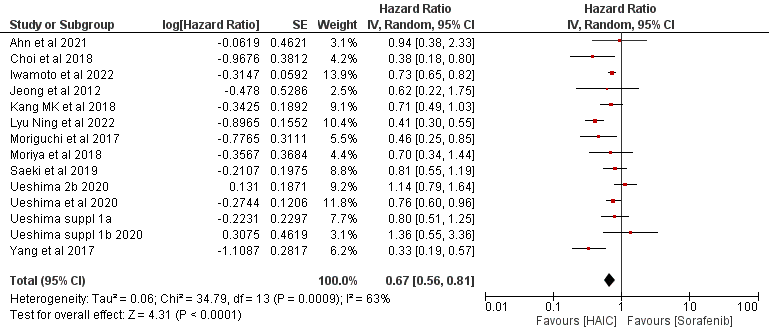
Supplementary Figure 9B Forest plot about the hazard ratio of overall survival (OS) in chemotherapy naïve HCC patients**

**
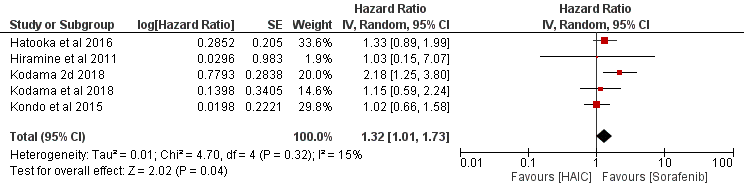
Supplementary Figure 9A Forest plot about the hazard ratio of overall survival (OS) in HCC patients who were refractory to TACE**

**Supplementary File 10A-D Subgroup analysis of overall survival based on tumour characteristic**

**
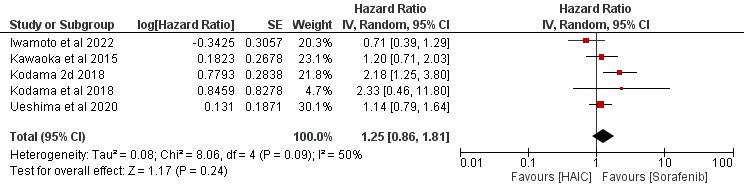
Supplementary Figure 10A Forest plot about the hazard ratio of overall survival (OS) in HCC patients without extrahepatic spread (EHS) or major vascular invasion (MVI)**

**Supplementary Figure 10B Forest plot about the hazard ratio of overall survival (OS) in HCC patients with extrahepatic spread (EHS) but no major vascular invasion (MVI)**

**
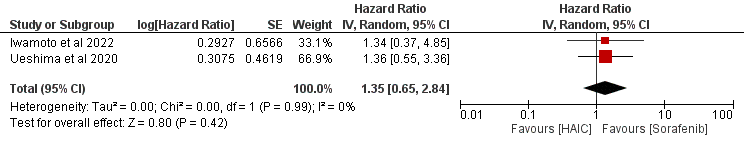
**

**
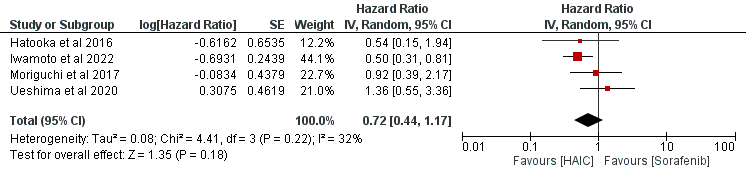
Supplementary Figure 10C Forest plot about the hazard ratio of overall survival (OS) in HCC patients with extrahepatic spread (EHS) and major vascular invasion (MVI)**

**
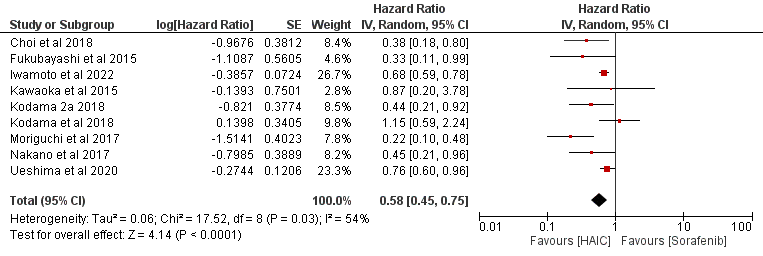
Supplementary Figure 10D Forest plot about the hazard ratio of overall survival (OS) in HCC patients with major vascular invasion (MVI) but no extrahepatic spread (EHS)**

**Supplementary File 11A-B Subgroup analysis of intermediate endpoint survival**

**Supplementary Figure 11A Forest plot about the hazard ratio of 1-year overall survival**

**
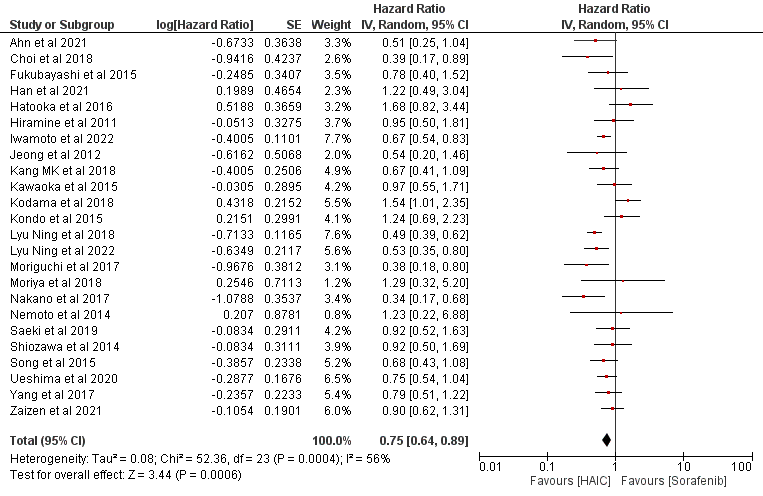
**

**Supplementary Figure 11B Forest plot about the hazard ratio of 1-year progression free survival**

**
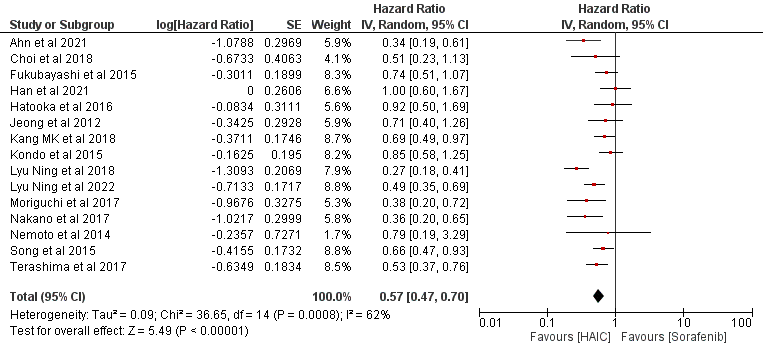
**

**Supplementary File 12A-D Subgroup analysis of overall survival based on propensity scoring matching**

**Supplementary Figure 12A Forest plot about the hazard ratio of overall survival (OS) (studies employed propensity scoring matching)**

**
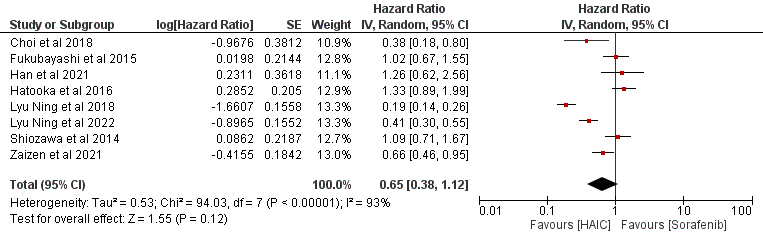
**

**Supplementary Figure 12B Forest plot about the hazard ratio of progression free survival (PFS) (studies employed propensity scoring matching)**

**
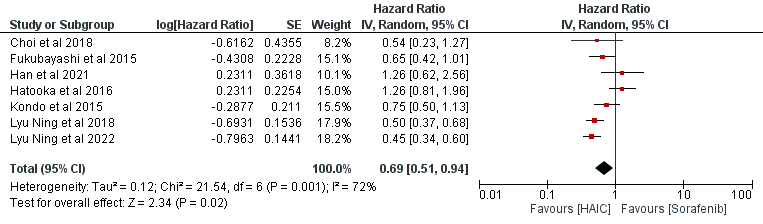
**

**Supplementary Figure 12C Forest plot about the hazard ratio of 1-year overall survival (1y-OS) (studies employed propensity scoring matching)**

**
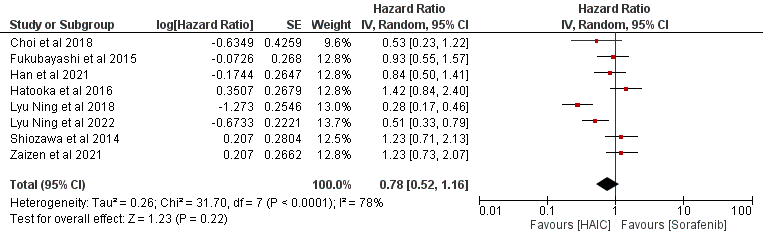
**

**Supplementary Figure 12C Forest plot about the hazard ratio of 1-year progression free survival (1y-PFS) (studies employed propensity scoring matching)**

**
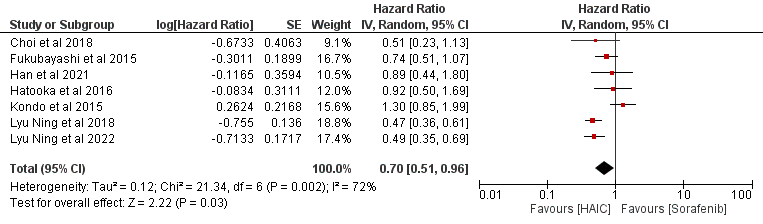
**

**Supplementary File 13A-M Grade III/IV Adverse Events**

**Supplementary Figure 13A Forest plot about the risk ratio of skin complications**

**
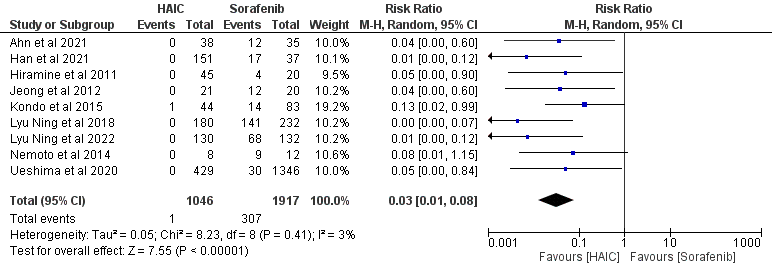
**

**Supplementary Figure 13B Forest plot about the risk ratio of elevated ALT**

**
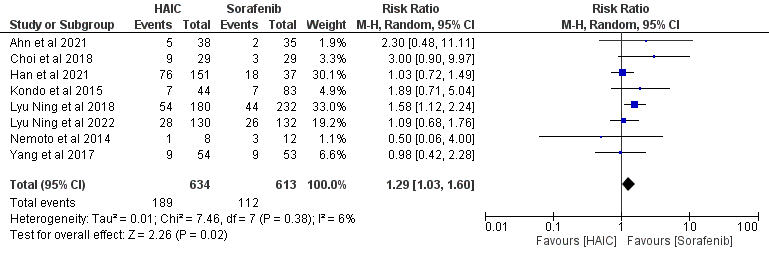
**

**Supplementary Figure 13C Forest plot about the risk ratio of diarrhoea**

**
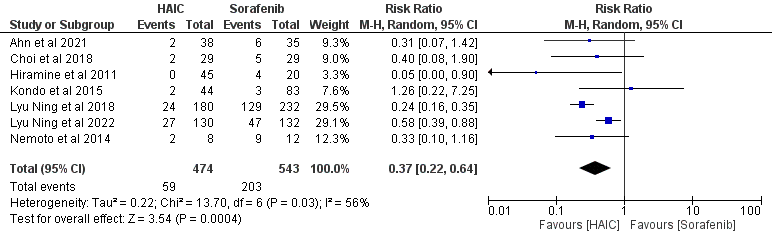
**

**Supplementary Figure 13D Forest plot about the risk ratio of ascites**

**
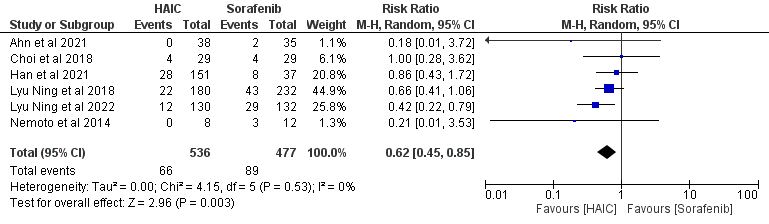
**

**Supplementary Figure 13E Forest plot about the risk ratio of neutropenia**

**
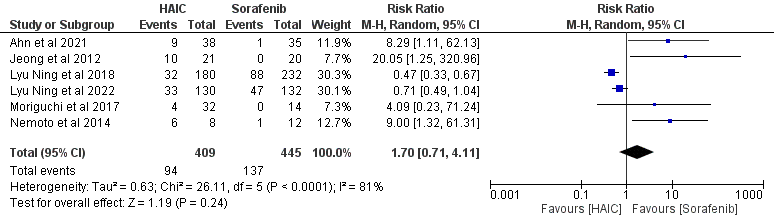
**

**Supplementary Figure 13F Forest plot about the risk ratio of hepatic encephalopathy**

**
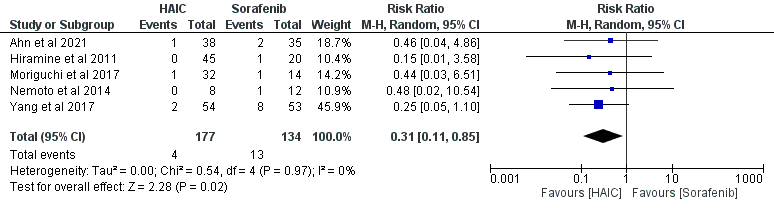
**

**Supplementary Figure 13G Forest plot about the risk ratio of elevated total bilirubin**

**
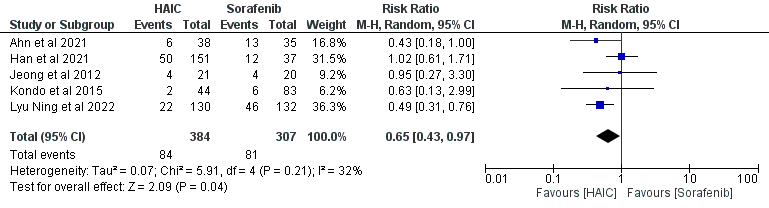
**

**Supplementary Figure 13H Forest plot about the risk ratio of elevated total bilirubin**

**
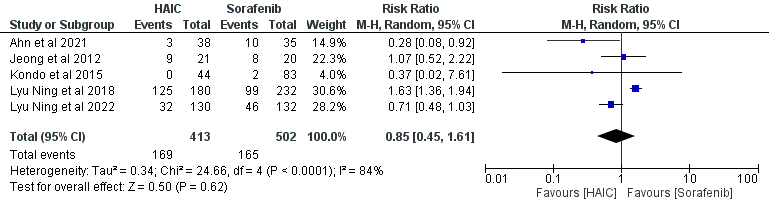
**

**Supplementary Figure 13I Forest plot about the risk ratio of thrombocytopenia**

**
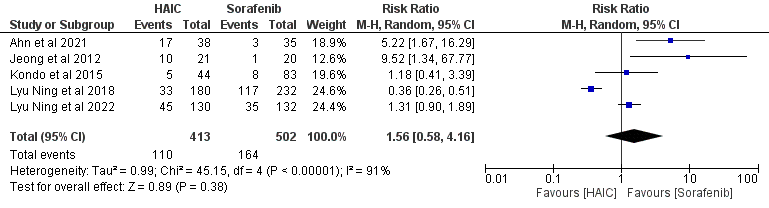
**

**Supplementary Figure 13J Forest plot about the risk ratio of leukopenia**

**
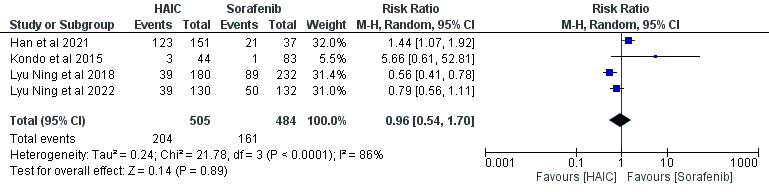
**

**Supplementary Figure 13K Forest plot about the risk ratio of reduced haemoglobin**

**
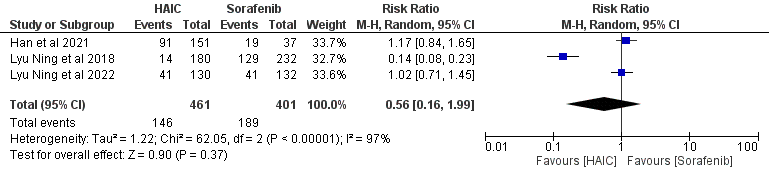
**

**Supplementary Figure 13L Forest plot about the risk ratio of hypoalbuminemia**

**
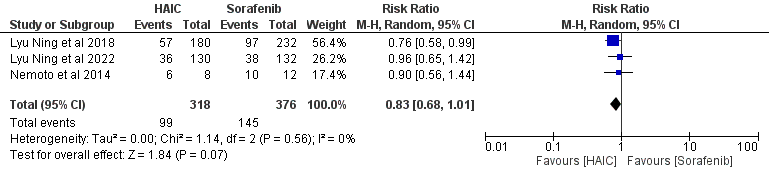
**

**Supplementary notation 13M: Definition of Grade III/IV adverse event**

**Grade III (Severe):** Adverse events classified as Grade III are considered severe. They typically result in substantial interference with daily activities, may require medical intervention or treatment modification, and can lead to hospitalization.

**Grade IV (Life-Threatening):** Adverse events classified as Grade IV are considered life-threatening. They pose an immediate risk to the patient's life and often require urgent medical attention. Without intervention, these events may result in death.

**Supplementary file 14: Funnel plots of all treatment effects**

**Funnel plot of progression free survival**

**Funnel plot of overall survival**

**
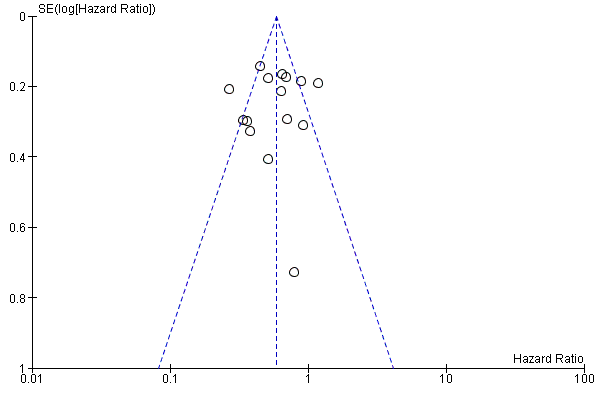

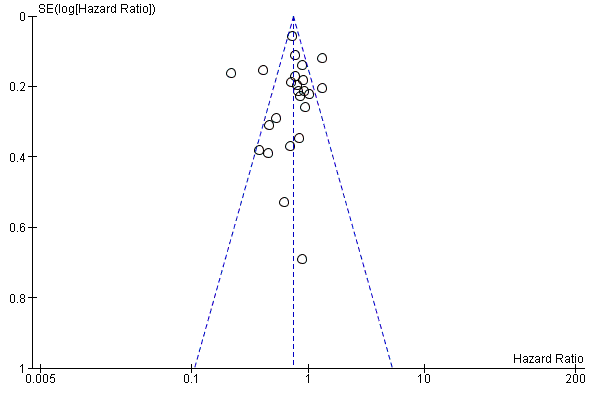
**

**
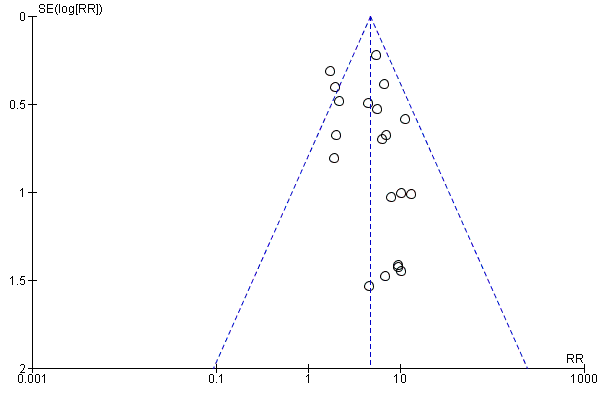

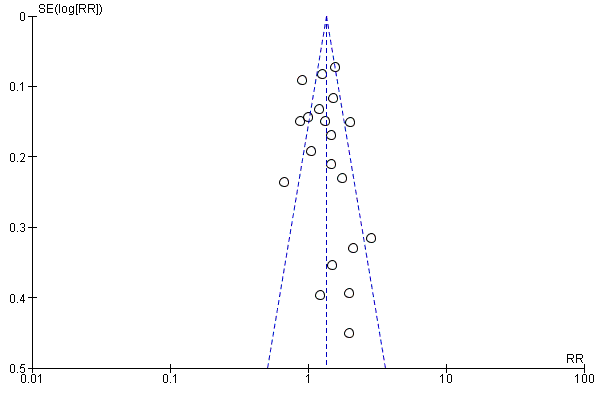

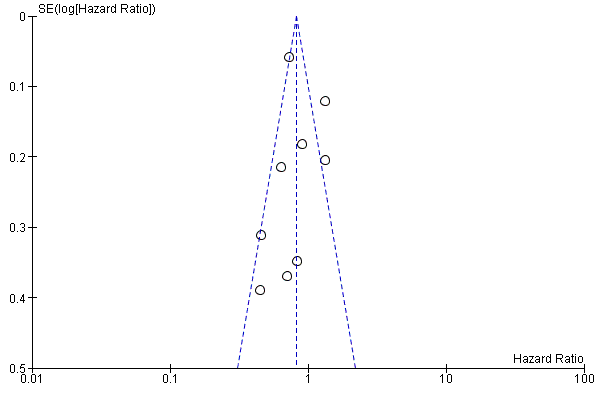

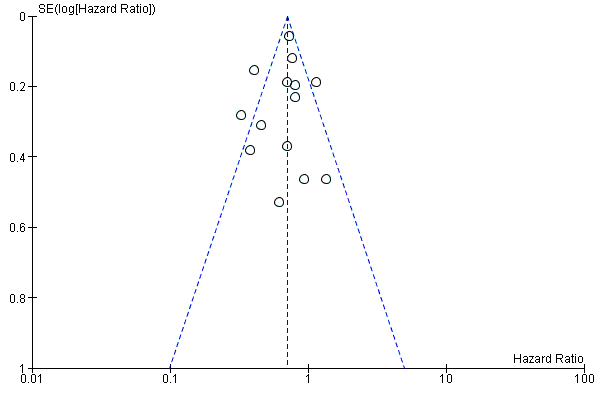
**

**Funnel plot of objective response rate**

**Funnel plot of disease control rate**

**Funnel plot of overall survival in Chemotherapy**

**naïve patients**

**Funnel plot of overall survival in Child-Pugh A patients**


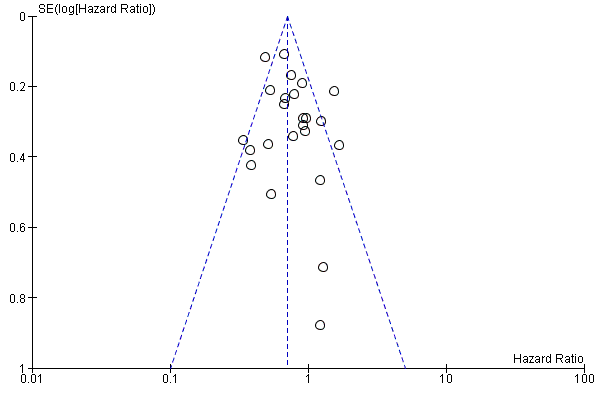

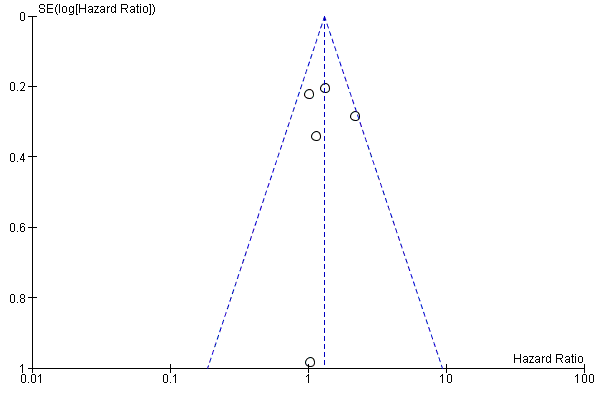


**Funnel plot of overall survival in patients with extrahepatic spread and major vascular invasion (EHS+MVI+)**

**Funnel plot of 1-year progression free survival**

**Funnel plot of overall survival in patients who were refractory to TACE**

**Funnel plot of 1-year overall survival**


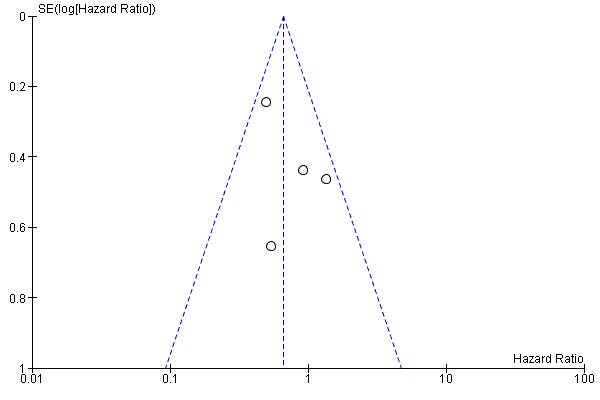

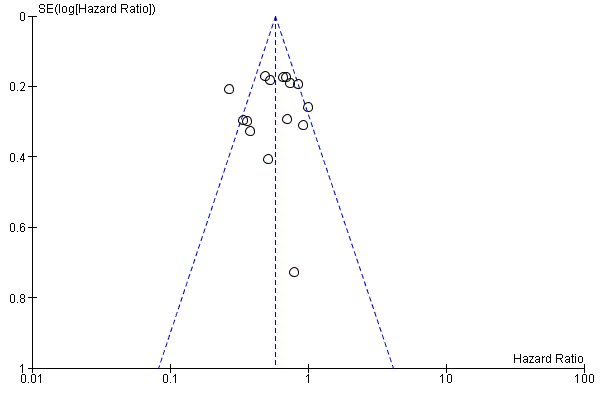


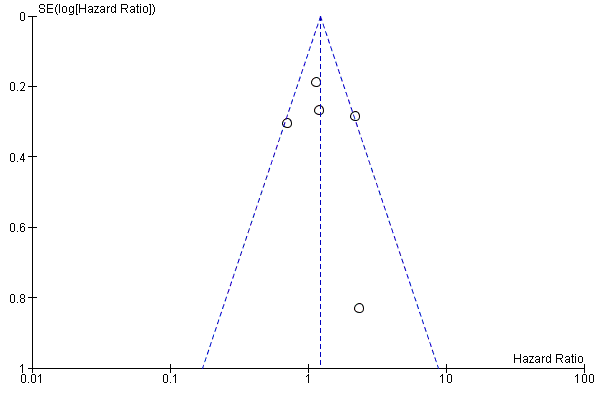


**Funnel plot of overall survival in patients without extrahepatic spread but had major vascular invasion (EHS-MVI+)**

**Funnel plot of overall survival in patients without extrahepatic spread or major vascular invasion (EHS-MVI-)**


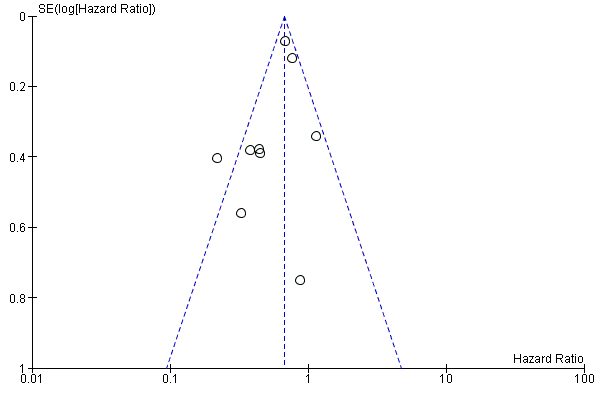


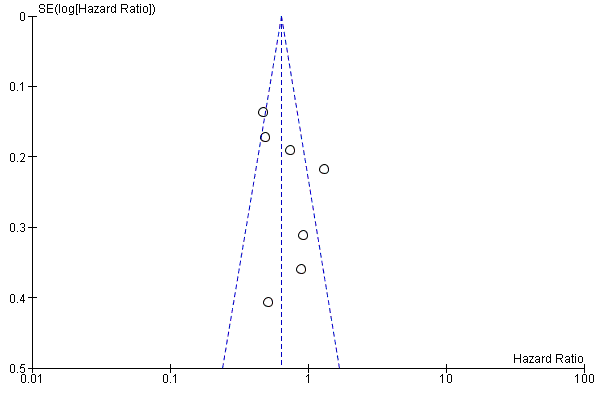

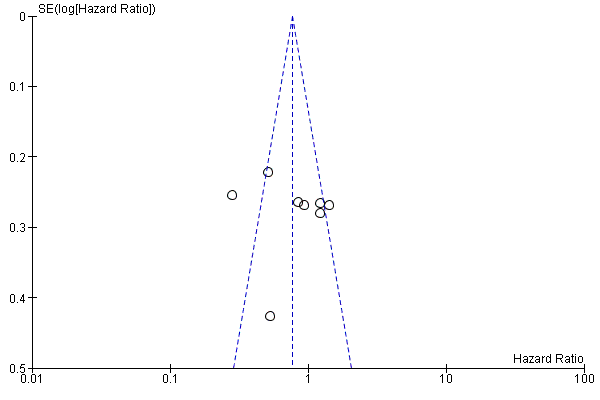
**
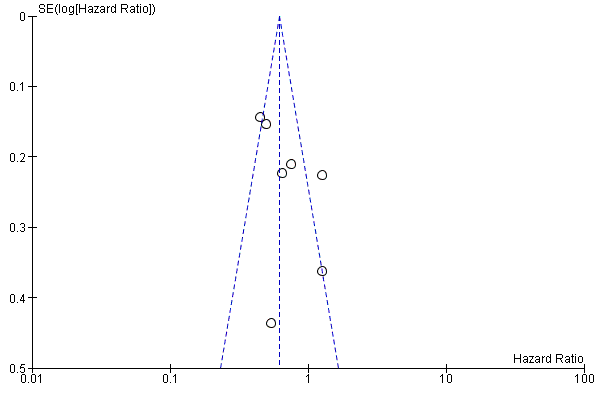
**
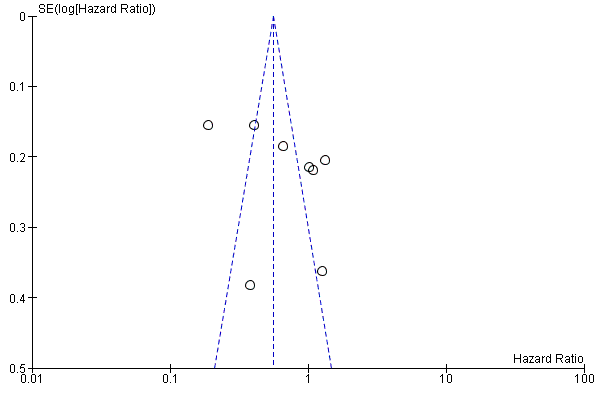


**Funnel plot of 1y-overall survival after propensity score matching**

**Funnel plot of 1y-progression free survival after propensity score matching**

**Funnel plot of progression free survival after propensity score matching**

**Funnel plot of overall survival after propensity score matching**

**Supplementary file 15A-L Potential risk and protective factors of overall survival**

**Supplementary Figure 15A Forest plot of multivariable HRs in Gender**


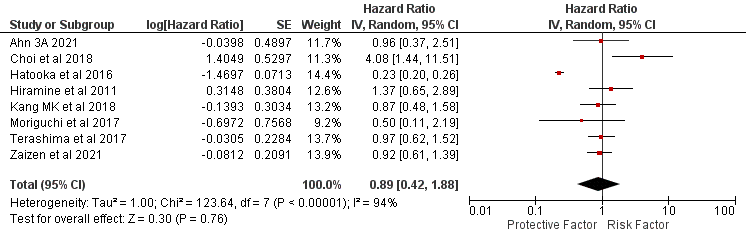


**Supplementary Figure 15B Forest plot of multivariable HRs in Age**


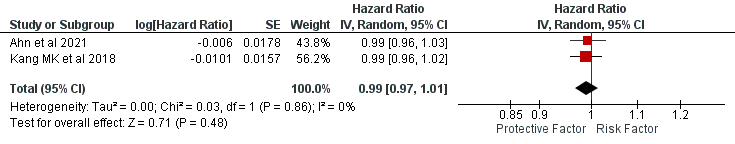


**Supplementary Figure 15C Forest plot of multivariable HRs in higher ECOG performance**

**
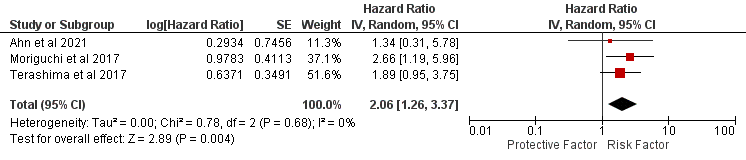
**

**Supplementary Figure 15D Forest plot of multivariable HRs in higher Child-Pugh Grade**

**
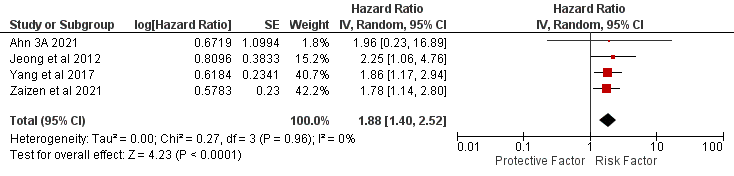
**

**Supplementary Figure 15E Forest plot of multivariable HRs in lower BCLC stage**


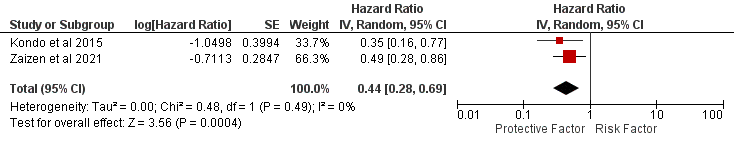


**Supplementary Figure 15F Forest plot of multivariable HRs in AFP(>400mg)**

**
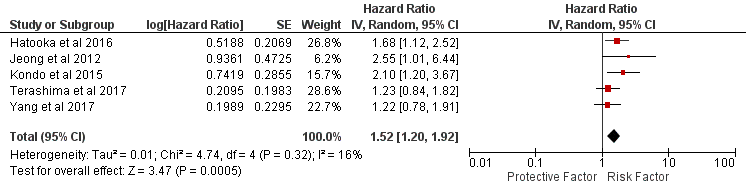
**

**Supplementary Figure 15G Forest plot of multivariable HRs in combination therapy**

**history**

**
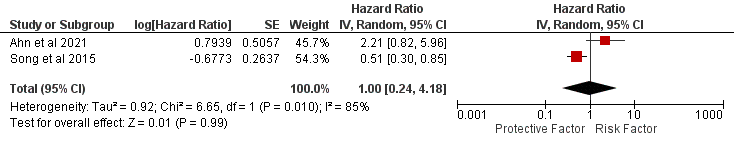
**

**Supplementary Figure 15H Forest plot of multivariable HRs in choosing HAIC therapy**

**
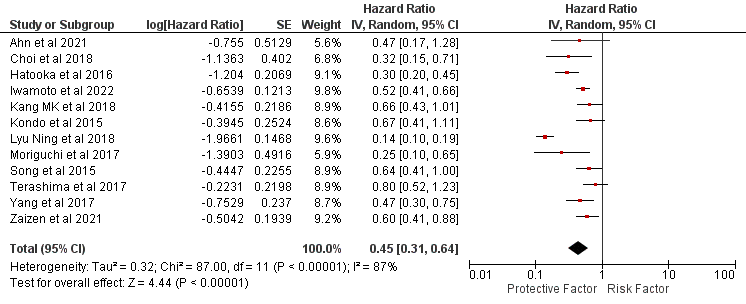
**

**Supplementary Figure 15I Forest plot of multivariable HRs in tumour burden (>50%)**

**
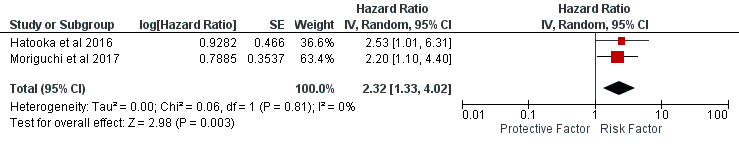
**

**Supplementary Figure 15I Forest plot of multivariable HRs in tumour diameter(>5cm)**

**
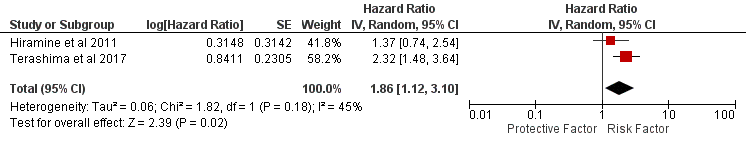
**

**Supplementary Figure 15J Forest plot of multivariable HRs in extrahepatic spread**

**
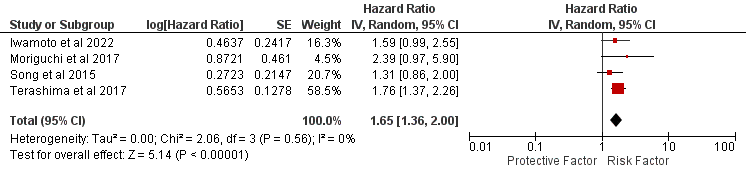
**

**Supplementary Figure 15K Forest plot of multivariable HRs in major vascular invasion**

**
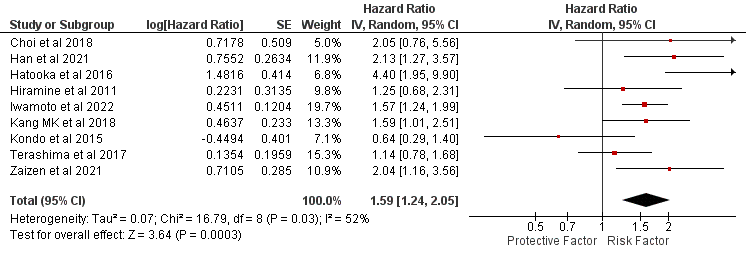
**

**Supplementary Figure 15L Forest plot of multivariable HRs in DCP (>400mAU/ml)**

**
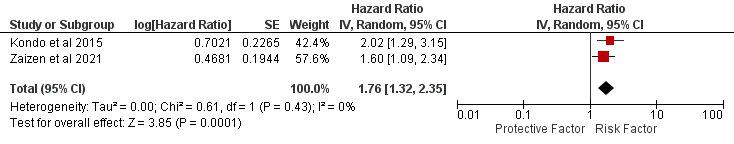
**

**Supplementary file 16 References from this study**

1. Zheng, K., et al., *Sorafenib Plus Hepatic Arterial Infusion Chemotherapy versus Sorafenib for Hepatocellular Carcinoma with Major Portal Vein Tumor Thrombosis: A Randomized Trial.* Radiology, 2022. **303**(2): p. 455-464.

2. Ueshima, K., et al., *Phase I/II Study of Sorafenib in Combination with Hepatic Arterial Infusion Chemotherapy Using Low-Dose Cisplatin and 5-Fluorouracil.* Liver Cancer, 2015. **4**(4): p. 263-73.

3. Reig, M., et al., *BCLC strategy for prognosis prediction and treatment recommendation: The 2022 update.* J Hepatol, 2022. **76**(3): p. 681-693.

4. Heimbach, J.K., et al., *AASLD guidelines for the treatment of hepatocellular carcinoma.* Hepatology, 2018. **67**(1): p. 358-380.

5. European Association for the Study of the Liver. Electronic address, e.e.e. and L. European Association for the Study of the, *EASL Clinical Practice Guidelines: Management of hepatocellular carcinoma.* J Hepatol, 2018. **69**(1): p. 182-236.

6. Chen, L.T., et al., *Pan-Asian adapted ESMO Clinical Practice Guidelines for the management of patients with intermediate and advanced/relapsed hepatocellular carcinoma: a TOS-ESMO initiative endorsed by CSCO, ISMPO, JSMO, KSMO, MOS and SSO.* Ann Oncol, 2020. **31**(3): p. 334-351.

7. Kudo, M., et al., *Management of Hepatocellular Carcinoma in Japan: JSH Consensus Statements and Recommendations 2021 Update.* Liver Cancer, 2021. **10**(3): p. 181-223.

8. Si, T., et al., *Hepatic arterial infusion chemotherapy versus transarterial chemoembolization for unresectable hepatocellular carcinoma: A systematic review with meta-analysis.* Front Bioeng Biotechnol, 2022. **10**: p. 1010824.

9. Lyu, N., et al., *Arterial Chemotherapy of Oxaliplatin Plus Fluorouracil Versus Sorafenib in Advanced Hepatocellular Carcinoma: A Biomolecular Exploratory, Randomized, Phase III Trial (FOHAIC-1).* J Clin Oncol, 2022. **40**(5): p. 468-480.

10. Llovet, J.M., et al., *Hepatocellular carcinoma.* Nat Rev Dis Primers, 2021. **7**(1): p. 6.

11. Page, M.J., et al., *The PRISMA 2020 statement: an updated guideline for reporting systematic reviews.* BMJ, 2021. **372**: p. n71.

12. Guyatt, G.H., et al., *GRADE: an emerging consensus on rating quality of evidence and strength of recommendations.* BMJ, 2008. **336**(7650): p. 924-6.

13. Tierney, J.F., et al., *Practical methods for incorporating summary time-to-event data into meta-analysis.* Trials, 2007. **8**: p. 16.

14. Kang, M.K., J.G. Park, and H.J. Lee, *Comparison of clinical outcomes between sorafenib and hepatic artery infusion chemotherapy in advanced hepatocellular carcinoma: A STROBE-compliant article.* Medicine (Baltimore), 2018. **97**(17): p. e0611.

15. Kodama, K., et al., *Comparison of clinical outcome of hepatic arterial infusion chemotherapy and sorafenib for advanced hepatocellular carcinoma according to macrovascular invasion and transcatheter arterial chemoembolization refractory status.* J Gastroenterol Hepatol, 2018. **33**(10): p. 1780-1786.

16. Ahn, Y.E., et al., *Comparison of Sorafenib versus Hepatic Arterial Infusion Chemotherapy-Based Treatment for Advanced Hepatocellular Carcinoma with Portal Vein Tumor Thrombosis.* Gut Liver, 2021. **15**(2): p. 284-294.

17. Abdelmaksoud, A.H.K., et al., *Hepatic arterial infusion chemotherapy in the treatment of advanced hepatocellular carcinoma with portal vein thrombosis: a case-control study.* Clin Radiol, 2021. **76**(9): p. 709 e1-709 e6.

18. Choi, J.H., et al., *Randomized, prospective, comparative study on the effects and safety of sorafenib vs. hepatic arterial infusion chemotherapy in patients with advanced hepatocellular carcinoma with portal vein tumor thrombosis.* Cancer Chemother Pharmacol, 2018. **82**(3): p. 469-478.

19. Moriguchi, M., et al., *Sorafenib versus Hepatic Arterial Infusion Chemotherapy as Initial Treatment for Hepatocellular Carcinoma with Advanced Portal Vein Tumor Thrombosis.* Liver Cancer, 2017. **6**(4): p. 275-286.

20. Song, D.S., et al., *A comparative study between sorafenib and hepatic arterial infusion chemotherapy for advanced hepatocellular carcinoma with portal vein tumor thrombosis.* J Gastroenterol, 2015. **50**(4): p. 445-54.

21. Yang, H., et al., *A comparative study of sorafenib and metronomic chemotherapy for Barcelona Clinic Liver Cancer-stage C hepatocellular carcinoma with poor liver function.* Clin Mol Hepatol, 2017. **23**(2): p. 128-137.

22. Nakano, M., et al., *Clinical effects and safety of intra-arterial infusion therapy of cisplatin suspension in lipiodol combined with 5-fluorouracil versus sorafenib, for advanced hepatocellular carcinoma with macroscopic vascular invasion without extra-hepatic spread: A prospective cohort study.* Mol Clin Oncol, 2017. **7**(6): p. 1013-1020.

23. Kawaoka, T., et al., *Comparison of hepatic arterial infusion chemotherapy versus sorafenib monotherapy in patients with advanced hepatocellular carcinoma.* J Dig Dis, 2015. **16**(9): p. 505-12.

24. Shiozawa, K., et al., *Comparison of Sorafenib and Hepatic Arterial Infusion Chemotherapy for Advanced Hepatocellular Carcinoma: A Propensity Score Matching Study.* Hepatogastroenterology, 2014. **61**(132): p. 885-91.

25. Nemoto, T., et al., *Comparison of hepatic arterial infusion chemotherapy and sorafenib in elderly patients with advanced hepatocellular carcinoma: A case series.* Mol Clin Oncol, 2014. **2**(6): p. 1028-1034.

26. Jeong, S.W., et al., *The efficacy of hepatic arterial infusion chemotherapy as an alternative to sorafenib in advanced hepatocellular carcinoma.* Asia Pac J Clin Oncol, 2012. **8**(2): p. 164-71.

27. Hiramine, Y., et al., *Sorafenib and hepatic arterial infusion chemotherapy for unresectable advanced hepatocellular carcinoma: A comparative study.* Exp Ther Med, 2011. **2**(3): p. 433-441.

28. Lyu, N., et al., *Hepatic arterial infusion of oxaliplatin plus fluorouracil/leucovorin vs. sorafenib for advanced hepatocellular carcinoma.* J Hepatol, 2018. **69**(1): p. 60-69.

29. Fukubayashi, K., et al., *Evaluation of sorafenib treatment and hepatic arterial infusion chemotherapy for advanced hepatocellular carcinoma: a comparative study using the propensity score matching method.* Cancer Med, 2015. **4**(8): p. 1214-23.

30. Hatooka, M., et al., *Comparison of Outcome of Hepatic Arterial Infusion Chemotherapy and Sorafenib in Patients with Hepatocellular Carcinoma Refractory to Transcatheter Arterial Chemoembolization.* Anticancer Res, 2016. **36**(7): p. 3523-9.

31. Ueshima, K., et al., *Hepatic Arterial Infusion Chemotherapy versus Sorafenib in Patients with Advanced Hepatocellular Carcinoma.* Liver Cancer, 2020. **9**(5): p. 583-595.

32. Terashima, T., et al., *Beneficial Effect of Maintaining Hepatic Reserve during Chemotherapy on the Outcomes of Patients with Hepatocellular Carcinoma.* Liver Cancer, 2017. **6**(3): p. 236-249.

33. Moriya, K., et al., *Efficacy of bi-monthly hepatic arterial infusion chemotherapy for advanced hepatocellular carcinoma.* J Gastrointest Oncol, 2018. **9**(4): p. 741-749.

34. Kondo, M., et al., *Hepatic arterial infusion chemotherapy with cisplatin and sorafenib in hepatocellular carcinoma patients unresponsive to transarterial chemoembolization: a propensity score-based weighting.* J Dig Dis, 2015. **16**(3): p. 143-51.

35. Zaizen, Y., et al., *Hepatic Arterial Infusion Chemotherapy with Cisplatin versus Sorafenib for Intrahepatic Advanced Hepatocellular Carcinoma: A Propensity Score-Matched Analysis.* Cancers (Basel), 2021. **13**(21).

36. Han, S., et al., *Treatment efficacy by hepatic arterial infusion chemotherapy vs. sorafenib after liver-directed concurrent chemoradiotherapy for advanced hepatocellular carcinoma.* J Cancer Res Clin Oncol, 2021. **147**(10): p. 3123-3133.

37. Saeki, I., et al., *Effect of body composition on survival benefit of hepatic arterial infusion chemotherapy for advanced hepatocellular carcinoma: A comparison with sorafenib therapy.* PLoS One, 2019. **14**(6): p. e0218136.

38. Iwamoto, H., et al., *The Clinical Impact of Hepatic Arterial Infusion Chemotherapy New-FP for Hepatocellular Carcinoma with Preserved Liver Function.* Cancers (Basel), 2022. **14**(19).

39. Liu, M., et al., *Systematic review of hepatic arterial infusion chemotherapy versus sorafenib in patients with hepatocellular carcinoma with portal vein tumor thrombosis.* J Gastroenterol Hepatol, 2020. **35**(8): p. 1277-1287.

40. Yan, L., et al., *A meta-analysis comparing hepatic arterial infusion chemotherapy and sorafenib for advanced hepatocellular carcinoma.* Transl Cancer Res, 2022. **11**(1): p. 99-112.

41. Zhang, W., et al., *Hepatic arterial infusion chemotherapy versus sorafenib for advanced hepatocellular carcinoma with portal vein tumor thrombus: An updated meta-analysis and systematic review.* Front Oncol, 2023. **13**: p. 1085166.

42. Ni, J.Y., et al., *Transcatheter hepatic arterial infusion chemotherapy vs sorafenib in the treatment of patients with hepatocellular carcinoma of Barcelona Clinic Liver Cancer stage C: a meta-analysis of Asian population.* Onco Targets Ther, 2018. **11**: p. 7883-7894.

43. Zhuang, B.W., et al., *Sorafenib versus hepatic arterial infusion chemotherapy for advanced hepatocellular carcinoma: a systematic review and meta-analysis.* Japanese Journal of Clinical Oncology, 2019. **49**(9): p. 845-855.

44. Si, T., et al., *Transarterial chemoembolization prior to liver transplantation for patients with hepatocellular carcinoma: A meta-analysis.* J Gastroenterol Hepatol, 2017. **32**(7): p. 1286-1294.

45. Song, M.J., *Hepatic artery infusion chemotherapy for advanced hepatocellular carcinoma.* World J Gastroenterol, 2015. **21**(13): p. 3843-9.

46. Lai, Z., et al., *Lenvatinib, toripalimab plus hepatic arterial infusion chemotherapy in patients with high-risk advanced hepatocellular carcinoma: A biomolecular exploratory, phase II trial.* Eur J Cancer, 2022. **174**: p. 68-77.

47. Zhang, T.Q., et al., *Camrelizumab (a PD-1 inhibitor) plus apatinib (an VEGFR-2 inhibitor) and hepatic artery infusion chemotherapy for hepatocellular carcinoma in Barcelona Clinic Liver Cancer stage C (TRIPLET): a phase II study.* Signal Transduct Target Ther, 2023. **8**(1): p. 413.

48. Kudo, M., et al., *Sorafenib plus low-dose cisplatin and fluorouracil hepatic arterial infusion chemotherapy versus sorafenib alone in patients with advanced hepatocellular carcinoma (SILIUS): a randomised, open label, phase 3 trial.* Lancet Gastroenterol Hepatol, 2018. **3**(6): p. 424-432.

49. He, M., et al., *Sorafenib Plus Hepatic Arterial Infusion of Oxaliplatin, Fluorouracil, and Leucovorin vs Sorafenib Alone for Hepatocellular Carcinoma With Portal Vein Invasion: A Randomized Clinical Trial.* JAMA Oncol, 2019. **5**(7): p. 953-960.

50. Kondo, M., et al., *Randomized, phase II trial of sequential hepatic arterial infusion chemotherapy and sorafenib versus sorafenib alone as initial therapy for advanced hepatocellular carcinoma: SCOOP-2 trial.* BMC Cancer, 2019. **19**(1): p. 954.

51. Long, Y., et al., *Sorafenib plus hepatic arterial infusion chemotherapy versus sorafenib alone for advanced hepatocellular carcinoma: A systematic review and meta-analysis.* J Gastroenterol Hepatol, 2023. **38**(4): p. 486-495.

52. Llovet, J.M., R. Montal, and A. Villanueva, *Randomized trials and endpoints in advanced HCC: Role of PFS as a surrogate of survival.* J Hepatol, 2019. **70**(6): p. 1262-1277.

53. Yang, J.D., et al., *A global view of hepatocellular carcinoma: trends, risk, prevention and management.* Nat Rev Gastroenterol Hepatol, 2019. **16**(10): p. 589-604.

54. Cheng, A.L., et al., *Updated efficacy and safety data from IMbrave150: Atezolizumab plus bevacizumab vs. sorafenib for unresectable hepatocellular carcinoma.* J Hepatol, 2022. **76**(4): p. 862-873.
